# Effect of temperature on structural properties and antibacterial performance of Fe–Co–Al@BTC MOF: A molecular docking and computational perspective

**DOI:** 10.1038/s41598-025-27018-5

**Published:** 2025-12-02

**Authors:** Eman Abdelnasser, Asmaa M. Fahim, Abdulrhman M. Alaraj, Mahmoud Abdelfatah, Abdelhamid El-Shaer

**Affiliations:** 1 Chemistry Department, Faculty of Science, Kafrelsheikh University, El-Geish Street, P.O. Box 33516, Kafrelsheikh, Egypt; 2 Department of Green Chemistry, National Research Centre, Dokki, P.O. Box.12622, Cairo, Egypt; 3 Physics Department, Faculty of Science, Kafrelsheikh University, Kafrelsheikh, 33516 Egypt; 4 Nano Science and Technology Program, Faculty of Science, Kafrelsheikh University, Kafrelsheikh, 33516 Egypt

**Keywords:** Antibacterial activity, Bacillus bacterial cells, Computational investigation, Docking investigation, Metal-organic frameworks (MOF), Temperature, Chemistry, Materials science

## Abstract

In this study, the impact of temperature on metal-organic framework (Fe-Co-Al @BTC) structural properties and antibacterial activity was investigated. It was synthesized by both hydrothermal method and at room temperature. It exhibited remarkable differences in crystallinity, porosity, morphology, and antibacterial activity. Fe-Co-Al@BTC prepared at room temperature exhibited higher crystallinity, larger average particle size, distinct morphology, and enhanced antibacterial activity compared to the hydrothermally synthesized sample. The estimated optical band gap was found to be ~ 2.48 eV and 2.25 eV for MOF synthesized at room temperature and hydrothermal conditions, respectively which was confirmed by PL results. Antibacterial performance, evaluated using optical density measurements and the cut plug method, demonstrated 100% bacterial growth inhibition at 600 mg/L for the room temperature sample, whereas the hydrothermal sample showed 50% inhibition at the same concentration. Density functional theory (DFT) calculations with the LANL2DZ basis set revealed the MOF’s electronic and photocatalytic properties, indicating stability, moderate reactivity, and potential for photocatalytic applications through analysis of the HOMO–LUMO gap and metal-to-ligand charge transfer. Thermodynamic analysis indicated that room temperature synthesis is more favorable despite slower crystallization, while hydrothermal synthesis is faster but energetically more demanding. Both syntheses were exothermic; however, higher temperature reduces spontaneity due to entropic penalties, with Gibbs free energy confirming room-temperature synthesis as the preferred approach.

## Introduction

Metal-organic frameworks (MOFs) are a unique class of crystalline porous materials constructed by self-assembling metal ions or clusters with organic linkers. Their high surface area, tunable pore size, and structural diversity make them attractive for various applications, including gas storage, catalysis, removal of heavy metals, sensing, drug delivery, and optoelectronics^1–9^. Temperature is a critical factor affecting the performance and stability of MOFs, as it can significantly alter their structural and functional properties. Temperature changes can significantly affect MOFs at multiple levels, including crystallinity, morphology, bonding interactions, optical properties, and porosity. At high temperatures, MOFs may undergo structural degradation, phase transitions, or partial decomposition of organic linkers, which in turn impacts their performance^2,10^.

To systematically investigate these effects, various characterization techniques are employed: X-ray diffraction (XRD) provides insights into the crystallographic structure and phase purity of MOFs. Changes in peak intensity, broadening, or disappearance indicate variations in crystallinity or framework collapse due to thermal treatment. Crystallinity is a fundamental structural property of MOFs that directly influences their functionality in applications such as catalysis, gas separation, antibacterial activity, drug delivery, and sensing. Changes in crystallinity directly affect reactivity and surface area. The highly ordered, periodic arrangement of metal nodes and organic linkers in crystalline MOFs ensures uniform pore structures and consistent physicochemical behavior^11^. However, temperature has a significant impact on MOF crystallinity. At moderate temperatures, thermal activation may improve crystallinity by removing trapped solvent molecules or unreacted ligands within the pores. In contrast, elevated temperatures can lead to thermal degradation, resulting in decreased crystallinity, partial amorphization, or complete structural collapse. These changes are commonly observed through X-ray diffraction (XRD) analysis, where high temperatures cause peak broadening, intensity reduction, or disappearance of characteristic diffraction peaks, indicating loss of long-range order. The extent of thermal stability and crystallinity retention depends on factors such as the strength of metal-ligand coordination, framework topology, and the presence of thermally labile components. Maintaining crystallinity under heat is essential for preserving the desirable properties of MOFs, especially in high-temperature applications^12^.

Raman spectroscopy enables analysis of molecular vibrations and bonding changes within the MOF structure. Temperature can influence metal-ligand coordination and symmetry, which are reflected in the shifting or diminishing of Raman bands^13,14^.

Surface area and pore size analysis, typically performed via nitrogen adsorption-desorption isotherms (BET method), is crucial for evaluating thermal effects on porosity. Heat can increase or decrease surface area and average pore size due to desorption of guest molecules or collapse of the framework^15,16^.

Since drug-resistant bacteria continually develop new resistance mechanisms, their spread increasingly threatens our ability to treat common diseases. The rapid global spread of multi- and pan-resistant bacteria, also known as “superbugs,” has alarmed the World Health Organization (WHO) because they can cause infections that are currently incurable with antimicrobial medications like antibiotics, antivirals, and antifungals^17,18^. Among these bacteria, *Bacillus* species, particularly *Bacillus cereus*, pose significant health concerns. While many *Bacillus* species are harmless, some can cause foodborne illnesses and opportunistic infections, especially in immunocompromised individuals. In Egypt, *Bacillus cereus* accounted for 46.6% of Bacillus isolates in hospital studies, followed by *Bacillus subtilis* at 38.1%^19^. Globally, the prevalence of *B. cereus* in food products is approximately 23.75%, with higher contamination rates in cereals, beans, vegetables, and dairy products^20^. The emergence of antimicrobial-resistant *Bacillus* strains highlights the urgent need for novel antibacterial agents, such as metal-organic frameworks (MOFs), to effectively combat these infections.

Metal–organic frameworks (MOFs) exhibit excellent antibacterial properties due to their unique structural and chemical features. Composed of metal ions or clusters coordinated to organic ligands, MOFs possess high surface area, tunable porosity, and the ability to incorporate bioactive metal centers such as silver (Ag), copper (Cu), and zinc (Zn). These metal ions are known for their inherent antimicrobial effects. MOFs can act directly by releasing toxic metal ions that disrupt bacterial cell membranes, interfere with intracellular functions, and generate reactive oxygen species (ROS), leading to oxidative stress and cell death. Additionally, MOFs can serve as carriers for antibiotics or antimicrobial agents, allowing for controlled and targeted drug release, enhancing the efficacy of treatment while minimizing side effects. Their structural versatility also allows for customization to target specific bacterial strains, making MOFs a promising class of materials in the fight against drug-resistant infections^21–23^. More than 20,000 MOF structures have been reported, covering different structural classes due to the different methods of formation, enhancing their applicability in fighting bacteria. The stability and porosity of MOFs can be improved through the incorporation of mixed linkers and bio-ligands such as amino acids and peptides for better biocompatibility^24^. External addition of metal ions or organic ligands, or the generation of free radicals, are various strategies offered by MOFs to exert antibacterial activities with the versatility for modifications. There is great interest in the treatment of bacterial infections with the use of integrated antibiotic alternatives as MOFs due to the presented problems of existing antibiotics^25^.

This study aims to comprehensively assess how temperature variations affect the structural integrity and physical properties of MOF by integrating these complementary techniques. Fe-Co-Al@BTC was synthesized by both hydrothermal and at room temperature. Antibacterial activity was employed to demonstrate the impact of temperature on the activity of MOF. The antibacterial activity was confirmed by docking simulations using PDBIDs: 4KIP and 6NMX, with RMSD = 0.89 and 0.93 Å, which demonstrates good structural stability and indicates that the MOF remains stable during the simulation. Moreover, computational studies revealed that Fe-Co-Al@BTC MOF exhibits stable electronic properties (HOMO-LUMO gap = 3.071 eV), moderate electrophilicity, and visible-light absorption (485–512 nm), making it suitable for photocatalysis and sensing. Room-temperature synthesis was thermodynamically favored (lower ΔG/ΔH), while hydrothermal synthesis accelerated crystallization but required more energy, with both methods being exothermic and structurally stable (RMSD < 1 Å).

## Materials and methods

### Chemicals

Table 1 summarizes the key information about the chemical reagents used in the experimental work. It lists the component names, empirical (molecular) formulas, molecular weights, suppliers, and purity levels of each chemical. All reagents were obtained from reputable suppliers, mainly Merck KGaA, except for N, N-dimethylformamide (DMF), which was supplied by Honeywell.

**Table 1:** Empirical Formula, CAS registry Number, Suppliers, and purity of the Chemicals.

Component	Empirical formula	Molecular weight (g/mol)	Suppliers	Purity (%)
1,3,5-Tricarboxybenzene	[C_6_H_3_(COOH)_3_ (BtC)]	210.14	Merck KGaA	≥ 95
Aluminum nitrate hexahydrate	AlN_3_O_9_·9H_2_O	375.13	Merck KGaA	≥ 95
Cobalt (II)nitrate hexahydrate	Co (NO_3_)_2_.6H_2_O	290.93	Merck KGaA	≥ 99
Iron (III) chloride nonahydrate	FeCl_3_.6H_2_O	404.00	Merck KGaA	≥ 99.5
Iron (III)nitrate nonahydrate	FeNO_3_.6H_2_O	349.951	Merck KGaA	≥ 99.5
Triethylamine	(C_2_H_5_)_3_N	101.191	Merck KGaA	> 99.8
N, N-dimethylformamide	C_3_H_7_NO	73.09	Honeywell	≥ 95

### Synthesis of (Fe-Co-Al@BTC MOF)

A. **Synthesized at room temperature**

Fe-Co-Al@BTC MOF was synthesized at room temperature (~ 27 °C). 1,3,5-benzenetricarboxylate (BTC) (0.01 mol, 2.10 g) was dissolved in dimethylformamide (DMF) in the presence of triethylamine (TEA). Subsequently, iron chloride (0.01 mol, 4.04 g), aluminum nitrate (0.01 mol, 3.75 g), and cobalt nitrite (0.01 mol, 2.90 g) were added to the solution under vigorous stirring for several hours (15 h). The resulting blackish-green precipitate was collected by filtration, washed repeatedly with DMF, and centrifuged to remove impurities. Finally, the product was dried in an oven at 60 °C to obtain the Fe–Co–Al@BTC MOF^26^.

B. **Synthesized by hydrothermal**

Fe-Co-Al@BTC MOF was prepared by the hydrothermal method. 1,3,5-benzenetricarboxylate (BTC, 0.01 mol, 2.10 g) was dissolved in dimethylformamide (DMF) in the presence of triethylamine (TEA). To this solution, iron nitrate (0.01 mol, 4.04 g), aluminum nitrate (0.01 mol, 3.49 g), and cobalt nitrite (0.01 mol, 2.90 g) were added with continuous stirring. The mixture was then transferred to a Teflon-lined stainless-steel autoclave and heated at ~ 120 °C for 24 h. After cooling to room temperature, the resulting red precipitate was collected by filtration, thoroughly washed several times with ethanol, methanol, and acetone to remove unreacted species and impurities, and finally dried for further use^27^.

### Instrumentation

X-ray diffraction (XRD) analysis was performed using a Shimadzu XRD-6000 at Kafrelsheikh University, recorded on a Pertpro diffractometer (Cu Kα1 radiation, 45 kV, λ = 1.5404 Å, 40 mA, USA) to study the structural properties of the synthesized Fe-Co-Al@BTC MOF. The transmission Electron Microscope 200 kV (Nanotechnology Institute, Kafr-Elsheikh University) was used to study the structure of the materials. The optical properties of Fe-Co-Al@BTC MOF were analyzed with a JASCOV-750 UV-Vis spectrophotometer. Infrared spectra, ranging from 400 to 4000 cm^− 1^, were obtained using a Thermo Fisher Scientific spectrometer (Waltham, MA) with KBr pellets. Raman spectra were recorded using a WITec alpha300 R system, and scanning electron microscopy (SEM) (JSM-6510LV, Kafrelsheikh University) was employed to analyze the morphological characteristics of the synthesized Fe-Co-Al@BTC metal-organic framework (MOF).

### Structural simulation and powder X-Ray diffraction analysis

In the computational study, the structure of the Fe-Co-Al @BTC MOF was constructed in the Reflex module in the program Materials Studio^28^. The structure determination was done in four steps and integrated within the Accelrys Materials Studio 7.0 software platform, which was focused on powder X-ray diffraction (PXRD) results. Firstly, the Reflex module was used to model PXRD patterns, and in these models, the P*212121* space group was chosen for the primitive models^29^. To accurately determine the crystal type and approximate the cell parameters, an approach that utilizes the X-Cell algorithm was employed, and this approach analyzed the peak positions in the powder pattern and provided indexing. The more detailed structure of the crystal then underwent refinement, which involved gradually changing the lattice parameters and the structure until a good correspondence between the observed and calculated patterns was reached, as evidenced by Rwp and Rp values. This method was able to provide a good framework to address the complex problem of understanding crystalline structure, and it was able to do so from both theoretical and experimental perspectives with empirical results^30,31^.

### Bacterial strain and culture conditions

*Bacillus cereus* was isolated from drainage water and molecularly identified using 16 S rRNA gene sequencing. *B. cereus* strain was grown on agar plates containing tryptic soy agar media^32^. These agar plates were re-streaked every seven days and kept at 4 °C until the time of injection.

### Molecular Docking studies

To elucidate the binding interactions and predict the binding affinity of Fe–Co–Al@BTC MOF with essential bacterial target proteins, molecular docking simulations were performed. This computational approach provides atomistic insights into the antibacterial mechanism of the MOF, extending beyond simple metal ion release. software^33^. Conformational analysis was carried out with a 0.01 Å RMS slope threshold, and energy minimization of the MOF structure was performed using the MMFF94x force field. Further validation was performed using the AutoDock Vina verification module^34^. The crystal structures of two key bacterial enzymes were retrieved from the Protein Data Bank (PDB): The Threonine synthase from the Crystal structure of mitogen-activated protein kinase 14 (P38-H5) complex with 2-(2-CHLOROPHENYL)-N-(5-(CYCLOPROPYLCARBAMOYL)−2-METHYLPHENYL)−1,3-THIAZOLE-5-CARBOXAMIDE PDBID:4KIP^35^, and Threonine synthase from *Bacillus subtilis* ATCC 6633 with PLP and APPA(PDBID: 6NMX)^36^. Ten distributed docking simulations were carried out using standard conditions, with verification based on the E shape and the layout of general parameters. Protein structures were prepared for docking by removing water, adding hydrogen, and computing partial charges with the AMBER10:EHT forcefield. The MOF structure was treated as rigid during all docking simulations. The binding site was determined based on the location of the native co-crystallized ligand within each protein structure. For each protein target, ten independent docking simulations were conducted using the London dG scoring function to generate pose ensembles, and then refinement was done using the GBVI/WSA dG scoring function to estimate binding affinities (kcal/mol). The resulting poses were validated by calculating the root-mean-square deviation (RMSD) of the atomic positions between subsequent iterations to ensure convergence.

### Computational procedures

Density Functional Theory (DFT) calculations were employed to investigate the electronic structure, reactivity, and thermodynamic properties of the reactants and the synthesized Fe–Co–Al@BTC framework. All calculations were carried out using Gaussian 09 W software^37^. Given the periodic complexity of the full MOF structure, a molecular cluster approach was adopted. A representative fragment of the Fe–Co–Al@BTC framework was constructed, consisting of a single 1,3,5-benzenetricarboxylate (BTC) linker coordinated to the corresponding metal centers (Fe, Co, and Al). To maintain proper valency, the terminal bonds of the truncated cluster were capped with hydrogen atoms. Geometry optimizations were performed using the Berny optimization algorithm, ensuring convergence toward the most stable configuration^38,39^. No symmetry constraints were applied during the geometry optimization. The wide-ranging vibrational modes were assigned using the Potential Energy Distribution (PED), determined by the Vibrational Energy Distribution Analysis (VEDA) program^50^.

## Results and discussion

###  Synthesis of trimetallic Fe–Co–Al@BTC Metal-Organic framework

**Scheme 1:**
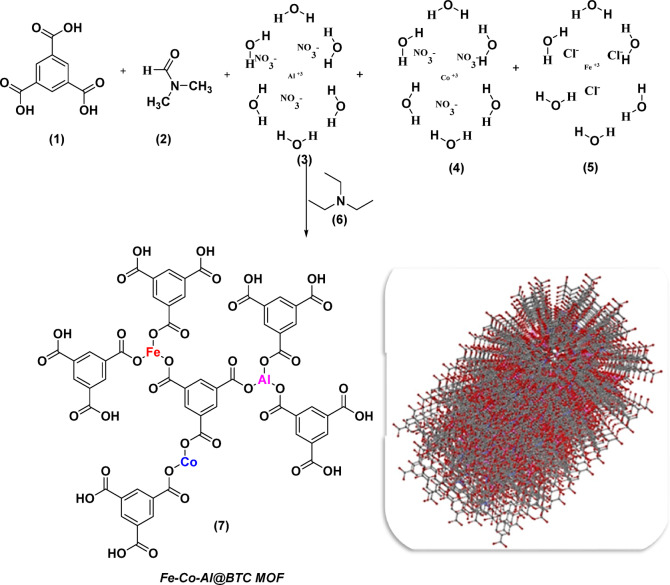
Outlines the essential steps involved in the synthesis of the Fe-Co-Al@BTC MOF, including reagent mixing and drying of the final product.

The trimetallic metal–organic framework (MOF), designated as Fe–Co–Al@BTC, was synthesized through a straightforward one-pot hydrothermal/room-temperature approach. Two synthetic strategies were employed to investigate the impact of temperature on the structural properties of the MOF. In this process, 1,3,5-benzenetricarboxylic acid (BTC) served as the organic linker, with a molar ratio of 1:1:1 for the metal precursors: aluminum nitrate nonahydrate [Al(NO₃)₃·9 H₂O], cobalt nitrate hexahydrate [Co(NO₃)₂·6 H₂O], and ferric chloride hexahydrate [FeCl₃·6 H₂O]. The reaction was conducted in N, N-dimethylformamide (DMF), a polar solvent, with triethylamine (TEA) added to deprotonate the carboxylic acid groups of BTC, thereby facilitating metal coordination. Upon mixing, BTC ligands chelated with Al³⁺, Co²⁺, and Fe³⁺ ions via their carboxylate groups, forming robust coordination bonds and driving the assembly of a three-dimensional porous MOF structure. The resulting material exhibited a uniform distribution of the three metal ions within the framework, with each contributing distinct functionalities: Fe³⁺ enhances redox activity, Co²⁺ imparts magnetic properties, and Al³⁺ reinforces structural integrity. The synergistic integration of these metal centers endowed Fe–Co–Al@BTC with superior physicochemical characteristics, positioning it as a versatile material with strong potential in catalysis, pollutant adsorption, and other environmental applications, as illustrated in Scheme 1.

### Effect of the temperature of formation of Fe-Co-Al@BTC MOF

**Fig. 1:**
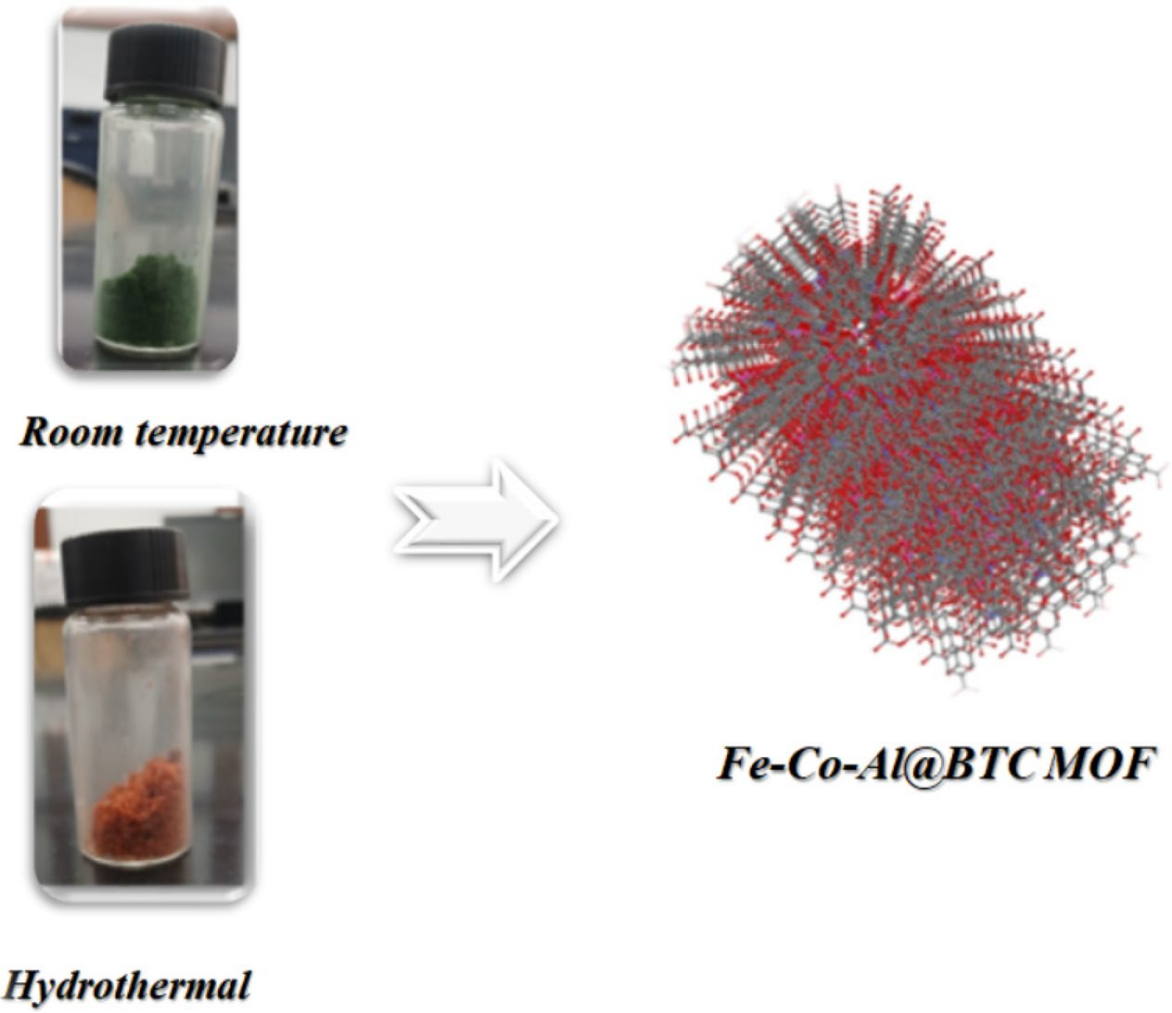
The formation of Fe-Co-Al@BTC MOF through thermal and room temperature.

Fe-Co-Al@BTC MOF can be prepared via different methods, including room temperature and hydrothermal. From Fig. 1, the green coloration at room temperature indicates potential differences in the metal oxidation state and coordination environment under mild conditions. The red coloration of the MOF indicates a different phase resulting from the metal-ligand interaction, and an enhanced metal oxidation process due to thermal action. Both differ in their physical and chemical properties.

#### FT-IR spectral analysis

**Fig. 2:**
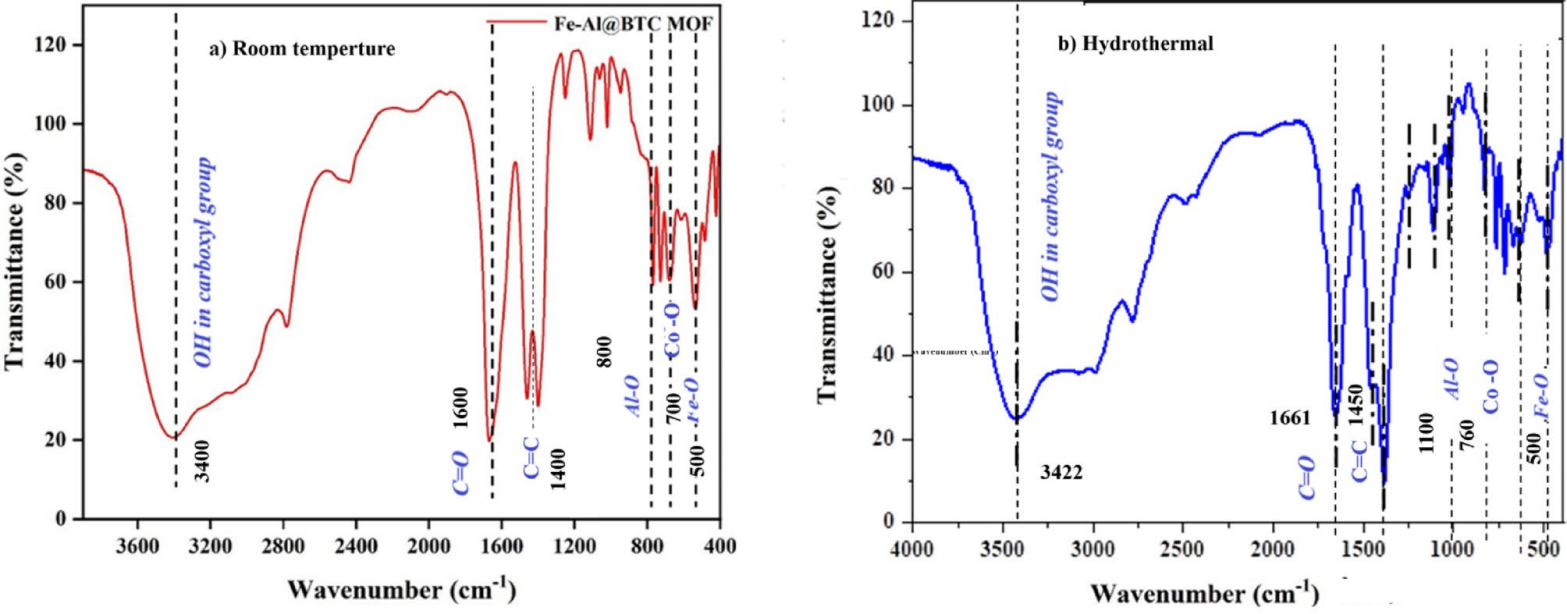
FT-IR spectral analysis of Fe-Co-Al@BTC MOF.

An essential aspect related to the structure of the Fe-Co-Al@BTC MOF is shown in Fig. 2 by its FT-IR spectrum. Absorption peak around 3400 and 3422 cm^− 1^ is owed to the presence of O-H functional groups in the metal hydroxides according to room temperature and hydrothermal methods^40^. The sharp peak located at 1661 and 1600 cm^− 1^ is related to C = O stretching vibrations, predicting attachment of the organic linker to the metal framework, contributing to the presence of the BTC linker according to room temperature and hydrothermal methods^41^. The peaks within the frequency range of 1450–1100 cm^− 1^ are attributed to the vibration of aromatic C = C bonds due to the benzene rings in the BTC linker, further illustrating the organic nature of the MOF^42^. A number of peaks in the 1000–500 cm^− 1^ range prove that the metal-organic coordination framework of iron-cobalt and the organic part of BTC has been synthesized successfully^43^. The less defined region of 500 cm^− 1^ and lower relative wave numbers show peaks that are associated with metal-ligand bonding vibration enhancing the formation of the Fe-Co-Al@BTC MOF^44^. It was insoluble in common organic solvents, such as acetone, methanol, ethanol, hexane, chloroform, tetrahydrofuran, dimethyl sulfoxide (DMSO), N, N-dimethylformamide (DMF), and water.

#### UV-visible analysis

**Fig. 3:**
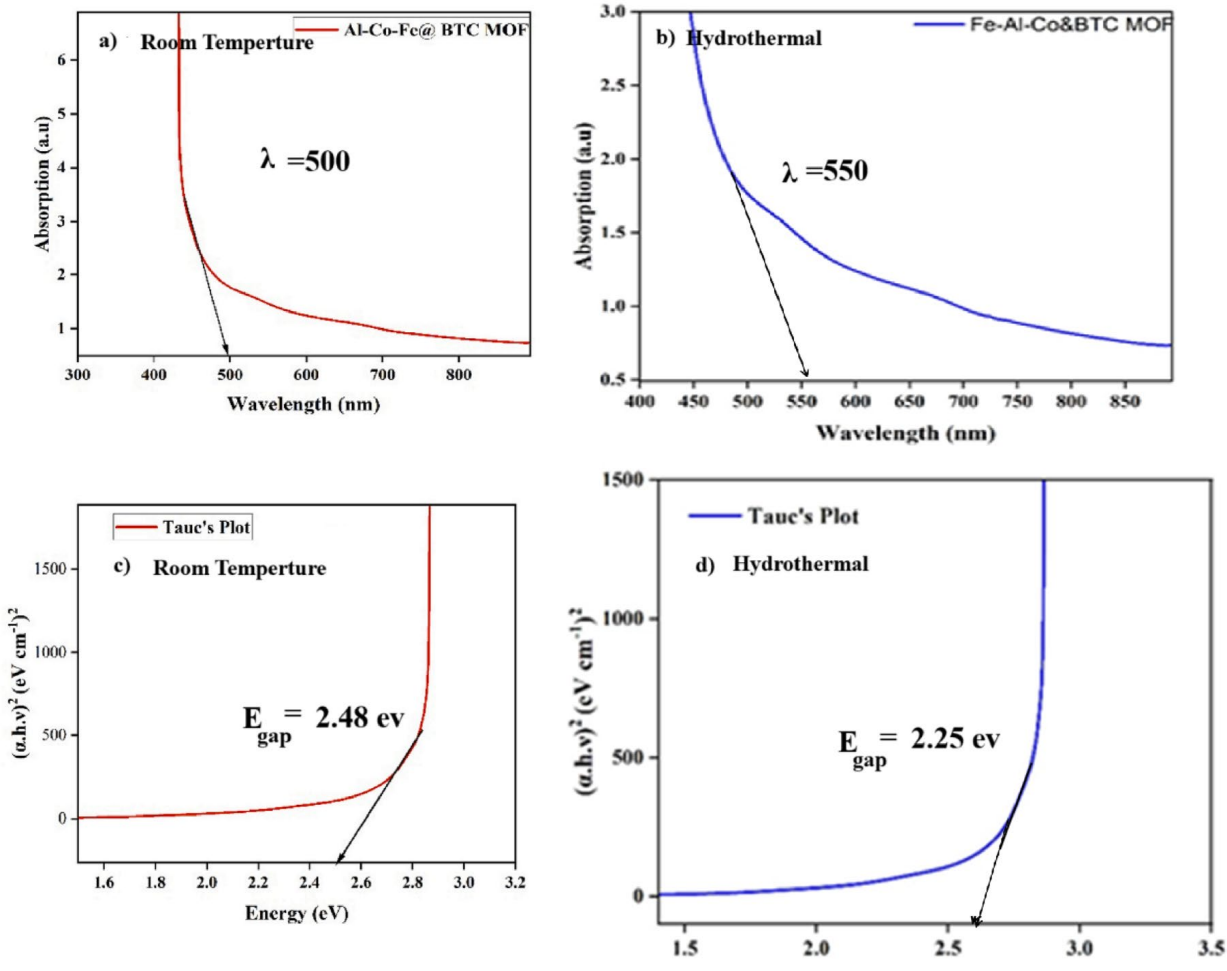
**(a)** UV-V spectrum of Fe-Co-Al@BTC MOF, **(b)** Tauc’s graph for the band gap measurement.

In Fig. 3, the UV–Vis spectra of the Al–Fe–BTC MOF demonstrate how the incorporation of cobalt atoms modifies the electronic structure of the material by introducing new energy levels and shifting the distribution of electronic states. This alteration impacts the band structure and results in a reduced band gap energy. From Tauc’s equation, (αhν)² = A(hν − E_gap_), the optical band gap was estimated to be ~ 2.29 eV, consistent with a direct allowed transition. Such modifications may arise from the formation of coordination complexes or covalent clusters within the MOF framework, leading to distinct optical characteristics (Table 2).

The absorption spectrum shows that the material strongly absorbs visible light in the 450–600 nm range. The Co ions in the MOF, similar to Fe ions, possess partially filled d-orbitals that enable d–d electronic transitions. Furthermore, the interaction between the d-orbitals of cobalt ions and the π-orbitals of the organic ligands promotes Ligand-to-Metal Charge Transfer (LMCT), while the reverse process induces Metal-to-Ligand Charge Transfer (MLCT). Thus, the incorporation of cobalt increases the number and probability of electronic transitions, enhancing visible-light absorption.

To further evaluate the photocatalytic potential of the material, the conduction band (CB) and valence band (VB) edge positions were calculated using the electronegativity method. Based on the absolute electronegativity of the constituent atoms, the CB edge was positioned at approximately − 0.14 V vs. NHE, while the VB edge was located at + 2.15 V vs. NHE. These values confirm that the conduction band is sufficiently negative to drive hydrogen evolution (H⁺/H₂, 0.00 V vs. NHE), while the valence band is more positive than the O₂/H₂O redox potential (+ 1.23 V vs. NHE), enabling water oxidation. Consequently, the band-edge alignment, combined with the narrowed band gap and extended visible-light absorption, demonstrates that the Co-doped Fe–Co–Al@BTC MOF exhibits favorable electronic properties for visible-light-driven photocatalysis^45^.

**Table 2:** Summmary table parametrs of Fe-Co-Al@BTC MOF.

Fe-Co-Al@BTC	Transition type	Eg (eV)	Assumed _χ_ (eV)	E_CB_ (eV vs. NHE	E_VB_ (eV vs. NHE)
Room temperature	Direct allowed	2.48 eV	5.6 eV	−0.14 eV	2.34 eV
Hydrothermal	Direct allowed	2.25 eV	5.6 eV	−0.19 eV	2.39 eV

#### Raman spectroscopy analysis

Raman spectroscopy enables analysis of molecular vibrations and bonding changes within the MOF structure. Temperature and synthesis conditions can influence metal–ligand coordination and framework symmetry, which are reflected in the shifting, broadening, or diminishing of Raman bands.

**Fig. 4:**
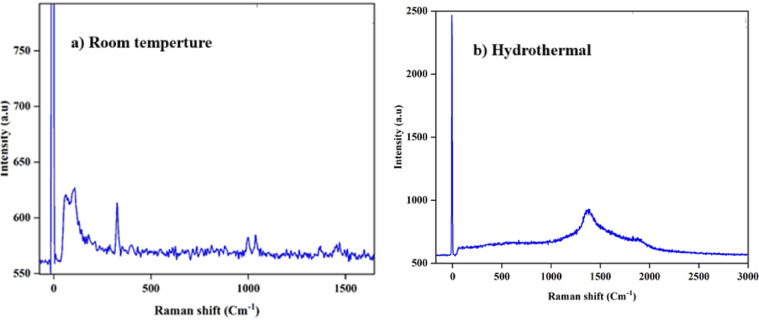
Spectral Raman Study of Fe-Co-Al@BTC Metal-Organic Framework.

For the Fe–Co–Al@BTC MOF synthesized at room temperature, the Raman spectrum (Fig. 4a) displays distinct vibrational bands, including features at approximately 200 cm⁻¹, 500 cm⁻¹, and 1000 cm⁻¹, which can be attributed to metal–oxygen stretching and carboxylate-related vibrations. In the 1000–1600 cm⁻¹ range, additional bands corresponding to aromatic C = C stretches from the BTC linker are clearly observed. The presence of sharp, well-defined peaks indicates successful coordination of the organic linker with the metal centers, confirming the crystalline nature of the MOF.

In contrast, for the hydrothermally synthesized sample (Fig. 4b), the Raman spectrum exhibits a broad hump centered around ~ 1500 cm⁻¹, without well-resolved peaks. This broadening suggests the loss of long-range order while maintaining only short-range metal–ligand coordination. Such behavior is characteristic of an amorphous phase, consistent with the corresponding XRD pattern, which also shows a diffuse halo rather than sharp Bragg reflections.

#### Powder X-ray diffraction of Fe-Al-Co@BTC MOF

**Fig. 5:**
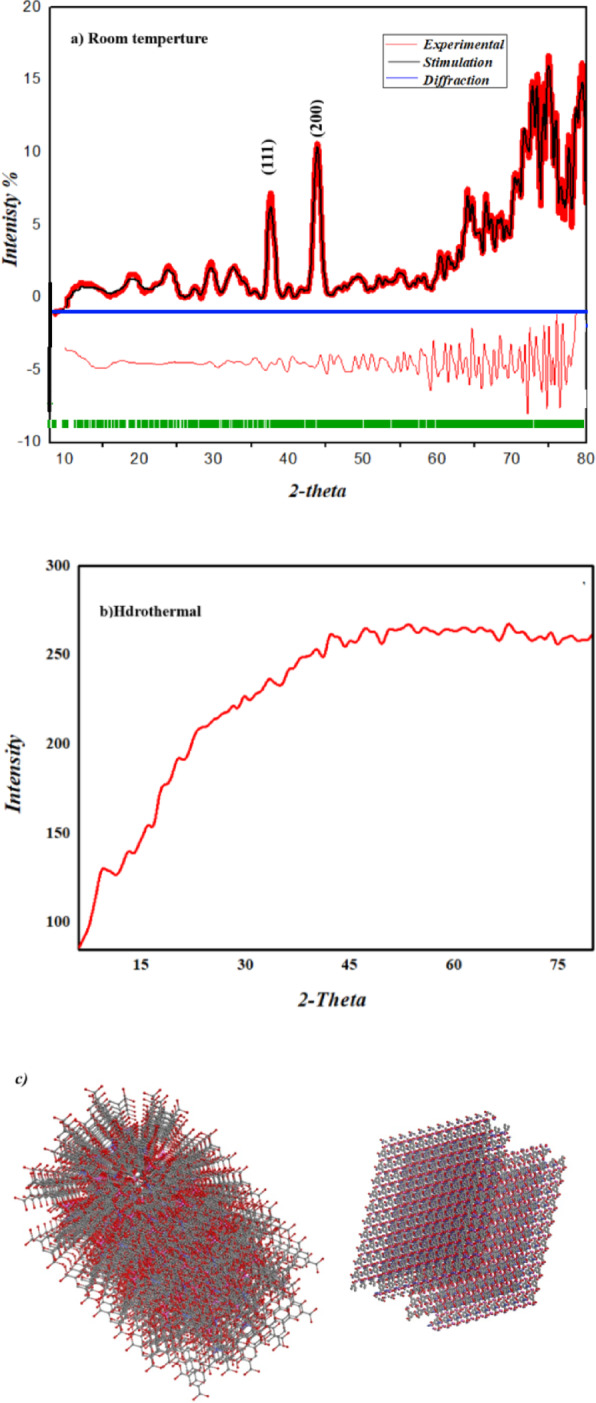
**(a)** XRD pattern for Fe-Co-Al@BTC MOF at room temperature; **(b)** XRD pattern at 120 °C, and **(c)** simulated structure of Fe-Co-Al@BTC MOF.

The computational analysis was conducted using *P21212* space group with α = β = γ = 90°, and unit cell dimensions of a = 16 Å, b = 13 Å, and c = 10 Å for the Fe-Co-Al @BTC MOF. These parameters closely matched the experimental structure. Furthermore, it is important that the Rwp = 8.19% and Rp = 6.31% factors allow us to conclude that the computed and the experimental intensity distributions are in good agreement. These parameters are essential in geochemistry and specifically in crystallography, where the lower the values, the better the observed data matches the computed data^46^ (Fig. 5a, b).

The crystal structure of the Fe-Co-Al@BTC MOF was analyzed using X-ray diffraction (XRD). The XRD pattern is shown in Fig. 5. showed that the Fe-Co-Al@BTC MOF has essentially no diffraction peak^47^, resulting in a broad diffraction feature due to the material’s short-range order. This short-range order can be caused by the existence of structural motifs, such as trinuclear metal nodes or clusters, that are repeated throughout the amorphous matrix but without a periodic pattern that extends over long distances^48^. According to previous studies, amorphous MOFs offer good electrochemical performance. Since it has been demonstrated that amorphous materials facilitate deeper electrolyte ion transport, this can significantly enhance the MOF electrode’s electrochemical characteristics due to its surface area^49^. By increasing the temperature of the reaction, it was found that the crystallinity of Fe-Co-Al@BTC MOF has been improved, as shown in Fig. 5b. The XRD pattern of c shows peaks at 2θ = 9.5, 37.6, 43.97, 64.2, and 77.3º, which are characteristic of the presence of Fe-Co-Al@BTC MOF.

#### Transmission electron microscopy (TEM)

**Fig. 6:**
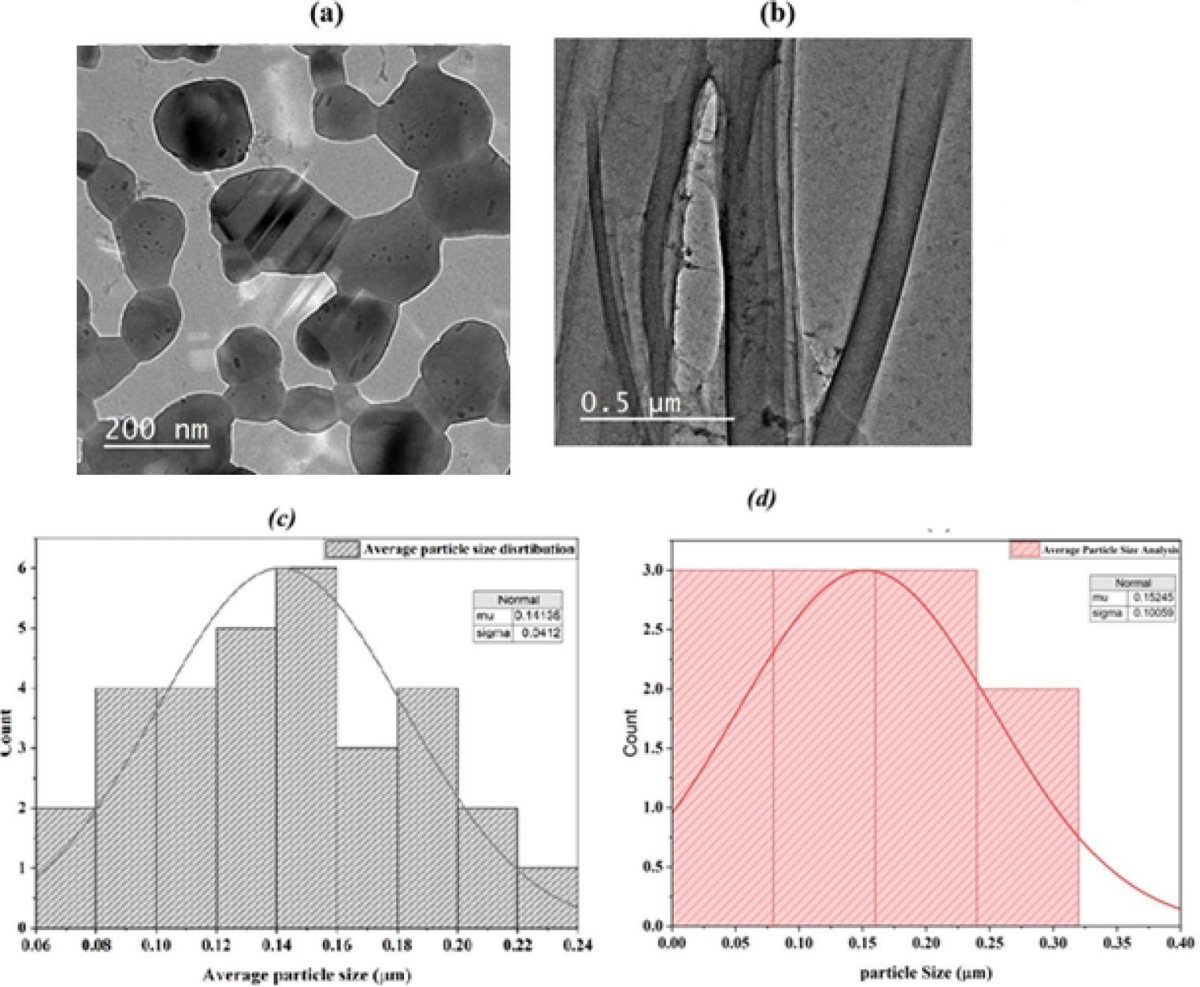
**(a**,** b)** TEM images of Fe-Co-Al@BTC MOF; **c)** Average particle size distribution of Fe-Co-Al@BTC MOF calculated using ImageJ software.

TEM image is demonstrated by the 0.5 μm scale bar in Fig. 6a, b as they are embedded in a fairly uniform matrix at a high magnification. The surface morphology of Fe-Co-Al@BTC at room temperature (Fig. 6a**)** was different from the surface morphology by hydrothermal (Fig. 6b**)**. The morphology at room temperature includes agglomerated clusters with a cauliflower-like texture. By hydrothermal, the morphology was rods, which differ in length, thickness, orientation, spacing, and consistency in surface pattern with minor defects. The image demonstrates the variation in material composition and density, with the rods being heavier. The rods all appear to be complete and porous, demonstrating good structural integrity. The mean particle size was determined as 0.14 and 0.15 μm at room temperature and hydrothermal conditions using ImageJ software as presented in Fig. 6c **d**.

#### Scanning electron microscope (SEM) & energy dispersive X-ray (EDX (analysis

**Fig. 7:**
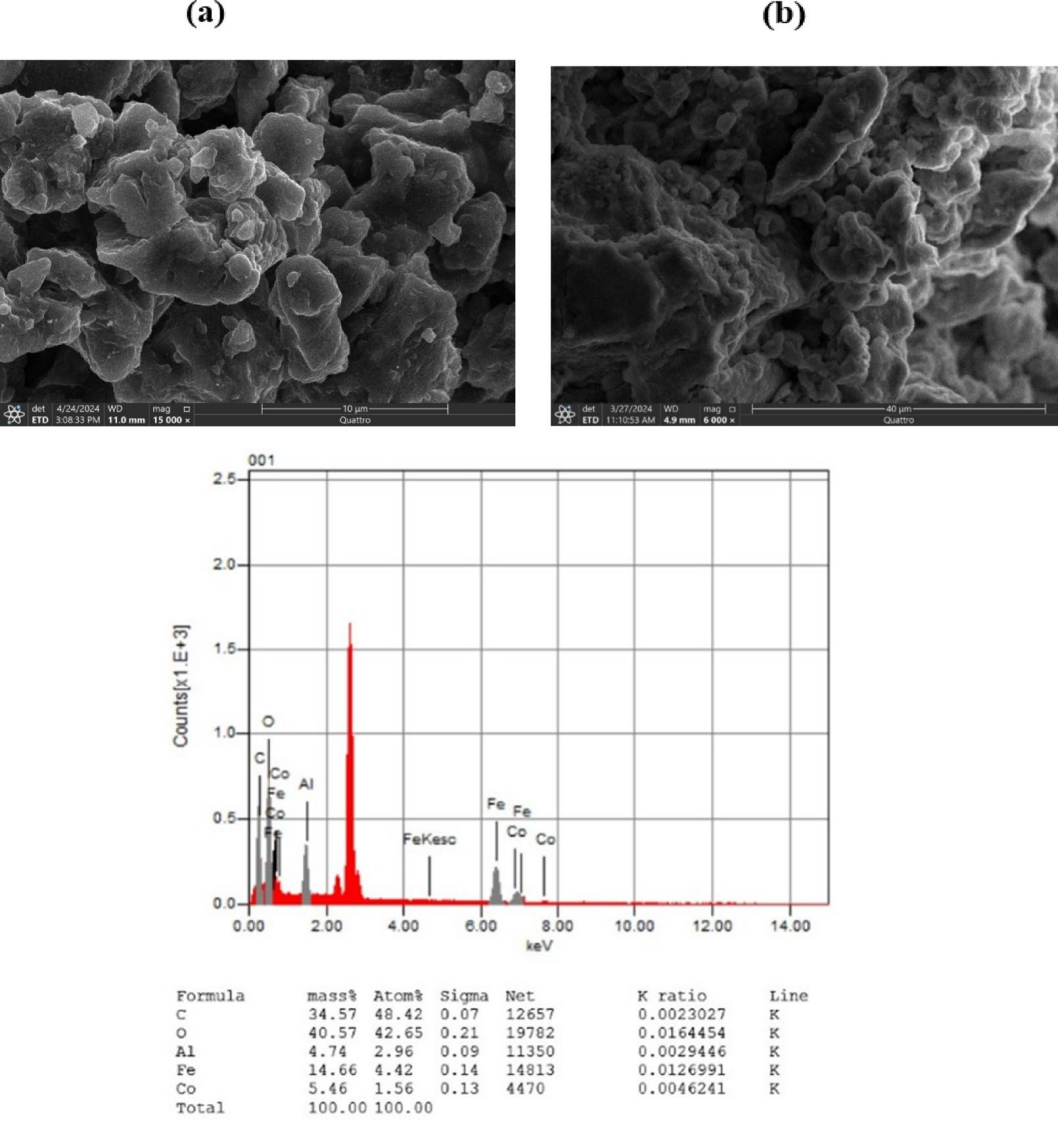
**(a**,** b)** Shows Scanning Electron Microscope images for Fe-Co-Al@BTC MOF **(c)** Energy Dispersive X-ray (EDX (analysis for Fe-Co-Al@BTC MOF.

The Scanning Electron Microscope (SEM) image offers an in-depth examination of the surface morphology of the Fe-Co-Al@BTC. It reveals a granular texture, with grains of varying sizes, suggesting a heterogeneous structure (Fig. 7a**).**. This granular appearance is characteristic of amorphous materials, as confirmed by X-ray Diffraction (XRD) analysis. Amorphous materials lack long-range order but may exhibit short-range structural motifs, such as trinuclear metal nodes or clusters (Fig. 7b**)**. These motifs are consistently repeated throughout the amorphous matrix, contributing to the overall structure without forming a periodic pattern. Energy Dispersive X-ray (EDX) analysis further supports these findings by identifying significant peaks for carbon and oxygen, which confirm the presence of organic linkers within the sample. Additionally, EDX detected iron (Fe), cobalt (Co), and aluminum (Al), indicating their successful incorporation into the MOF. Based on the EDX data, the empirical formula for the Fe-Al@BTC MOF is approximately C₃₄.₅₇, O₄₀.₅₇, Al₁₀.₀₉., Fe₁₀.₀₂, and Co₅.₄₆ as demonstrated in Fig. 7c.

### Photoluminescence analysis (PL)

**Fig. 8:**
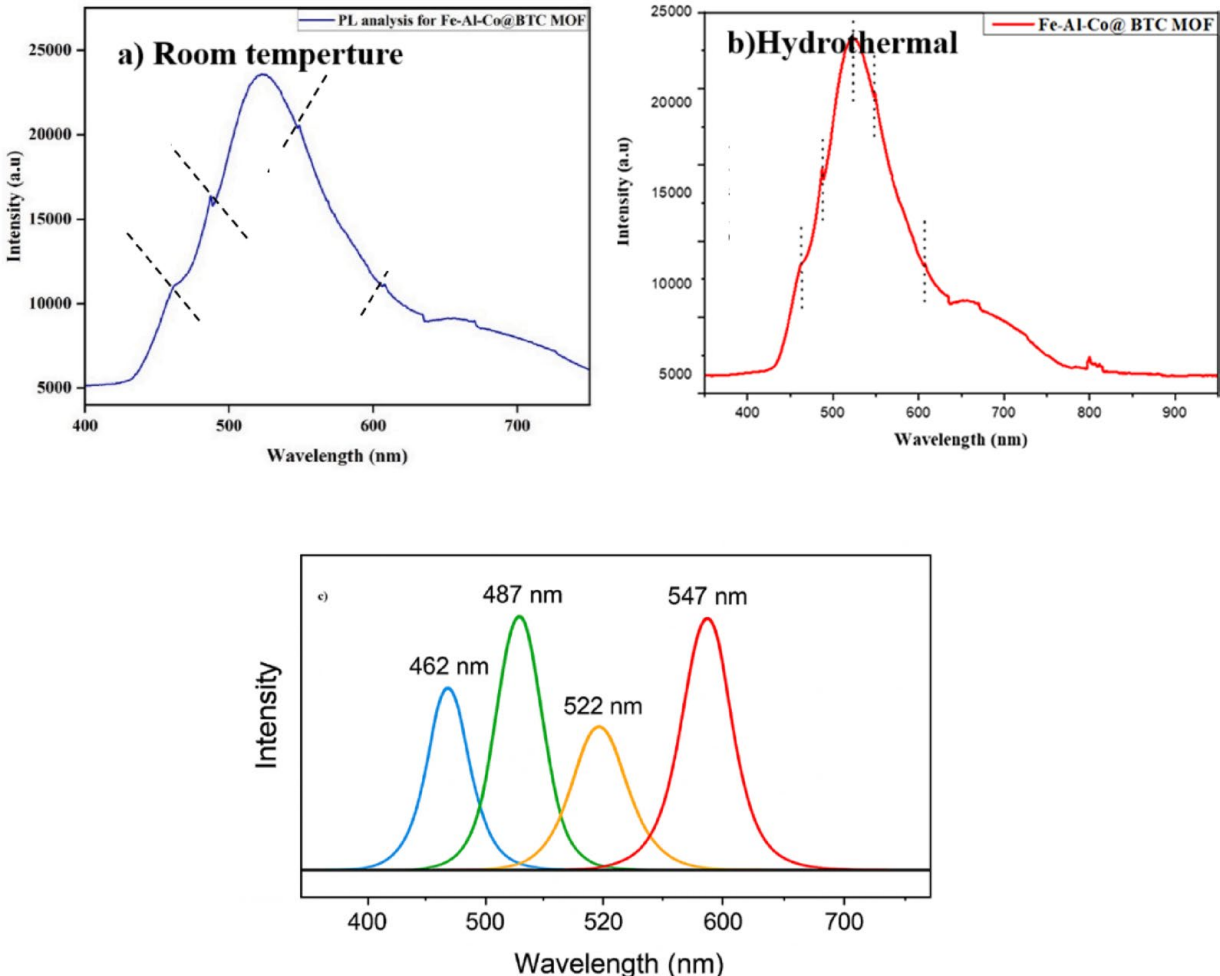
Photoluminescence spectra of Fe-Co-Al@ BTC MOF.

In the Co–Al–Fe@BTC MOF composite, the photoluminescence (PL) spectrum displayed peaks at 462, 487, 522, 547, and 607 nm (Fig. 8a, b). The blue-region peaks at 462 nm and 487 nm are attributed to excited states of the metal ions (Co, Al, Fe) or BTC linker transitions, possibly involving ligand-to-metal (LMCT) or metal-to-ligand (MLCT) charge transfer^50^. The green-region peaks at 522 nm and 547 nm may arise from intra-ligand transitions or defect-state emissions, whereas the red-region peak at 607 nm likely originates from deep trap states, Fe d–d transitions, or charge carrier recombination associated with structural defects^51^.

To provide a clearer understanding of these emissions, the PL spectrum was deconvoluted into individual Gaussian peaks (Fig. 8c). Each peak is labeled with its corresponding wavelength and color region, allowing precise assignment to specific electronic transitions, energy transfer processes between metal centers, and ligand–metal interactions^43^. This deconvoluted analysis offers a more detailed visualization of the contributions from different transitions and defect states within the MOF framework.

## ADME studies

**Fig. 9:**
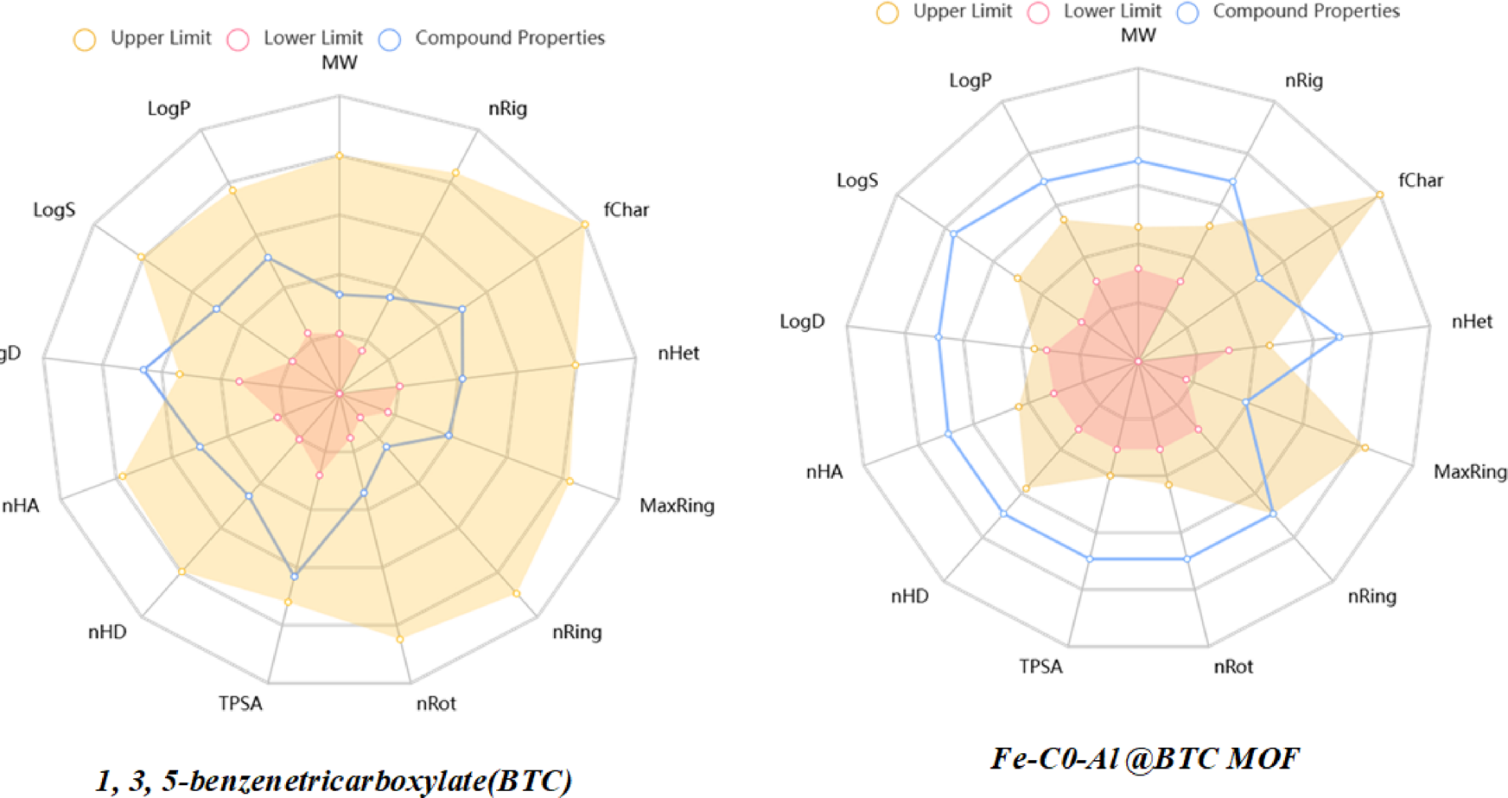
Bioavailability analysis of physicochemical properties by Swiss ADME radar.

Computational analysis serves as a critical first step in the drug discovery process, offering valuable insights that guide subsequent biological investigations. Advanced computer algorithms are utilized to evaluate the structural and behavioral characteristics of synthesized heterocyclic compounds. The physicochemical properties of these compounds play a pivotal role in determining their pharmacodynamic and pharmacokinetic profiles. The successful development of pharmaceuticals and industrial chemicals relies heavily on a comprehensive understanding of a molecule’s absorption, distribution, metabolism, excretion, and toxicity (ADME) characteristics. These properties are fundamental in predicting the compound’s efficacy, safety, and overall suitability for practical application. The drug-likeness of the phytochemicals was evaluated based on key physicochemical parameters obtained from the PubChem database and analyzed using the Swiss ADME web tool. The drug-like properties of phytochemicals derived from reactants and MOF compounds were assessed by Lipinski’s Rule of Five, a widely accepted guideline for evaluating the oral bioavailability of potential drug candidates. A demonstrated in Fig. 9; Table 3 the comparative analysis of key physicochemical parameters for the organic linker 1,3,5-benzenetricarboxylate (BTC) and the synthesized Fe-Co-Al@BTC metal-organic framework (MOF) and showed difference in behavior, solubility, and interaction potential of these materials in various environments. A higher log P value (4.852 for the MOF) implies greater lipid solubility, which can affect membrane permeability. Furthermore, Log D (Distribution Coefficient): Represents the ratio of a compound’s concentration between organic and aqueous phases, accounting for both ionized and unionized forms at a specific pH. The MOF exhibits a notably high Log D value of 18.407, indicating a strong preference for partitioning into the organic phase, and Flexibility: Reflects the molecular flexibility, typically associated with the number of rotatable bonds. The BTC ligand demonstrates lower flexibility with a value of 0.333, indicating a more rigid structure^52^. In contrast, the MOF exhibits greater flexibility (0.63), likely attributable to its more complex and extended framework^53^. So, we concluded the BTC ligand, characterized by its relatively low molecular weight and moderate lipophilicity, demonstrates limited structural flexibility and a balanced capacity for hydrogen bonding. Its topological polar surface area (TPSA) and log S values indicate favorable aqueous solubility and potential for effective surface interactions. In contrast, the Fe-CO-Al@BTC metal-organic framework (MOF) exhibits a markedly higher molecular weight and pronounced lipophilicity (log *P* = 4.852), along with an exceptionally high distribution coefficient (log D), reflecting a strong preference for nonpolar environments. MOF’s significantly elevated number of hydrogen bond donors and acceptors, along with its expansive TPSA and high molar refractivity, suggest a highly polar and interactive surface well-suited for guest molecule encapsulation or catalytic activity. Moreover, the enhanced structural flexibility of the MOF may further support dynamic host-guest interactions, contributing to its functional versatility in advanced applications.

**Table 3:** Physicochemical properties of 1,3,5-benzenetricarboxylate (BTC) and Fe-CO-Al@BTC.

Compound Number	1, 3, 5-benzenetricarboxylate (BTC)	Fe-CO-Al @BTC MOF
***Molecular weight (g/mol***	C_9_H_6_O_6_ (210.020 g/mol)	C_43_H_23_AlCoFeO_26_ (1393.890 g/mol)
***Lipophilicity (log p***	0.18	4.852
***Log D***	4.21	18.407
***Flexibility***	0.333	0.63
***Hydrogen bond donors (HD)***	3	10
***Hydrogen bond acceptors***	6	36
***TPSA (Å2)***	111.90 Å²	583.400 Å²
***Log S***	−1.56	5.010
***Molar Refractivity***	47.32	211.87

## Antimicrobial activities

### Antibacterial activity

**Fig. 10:**
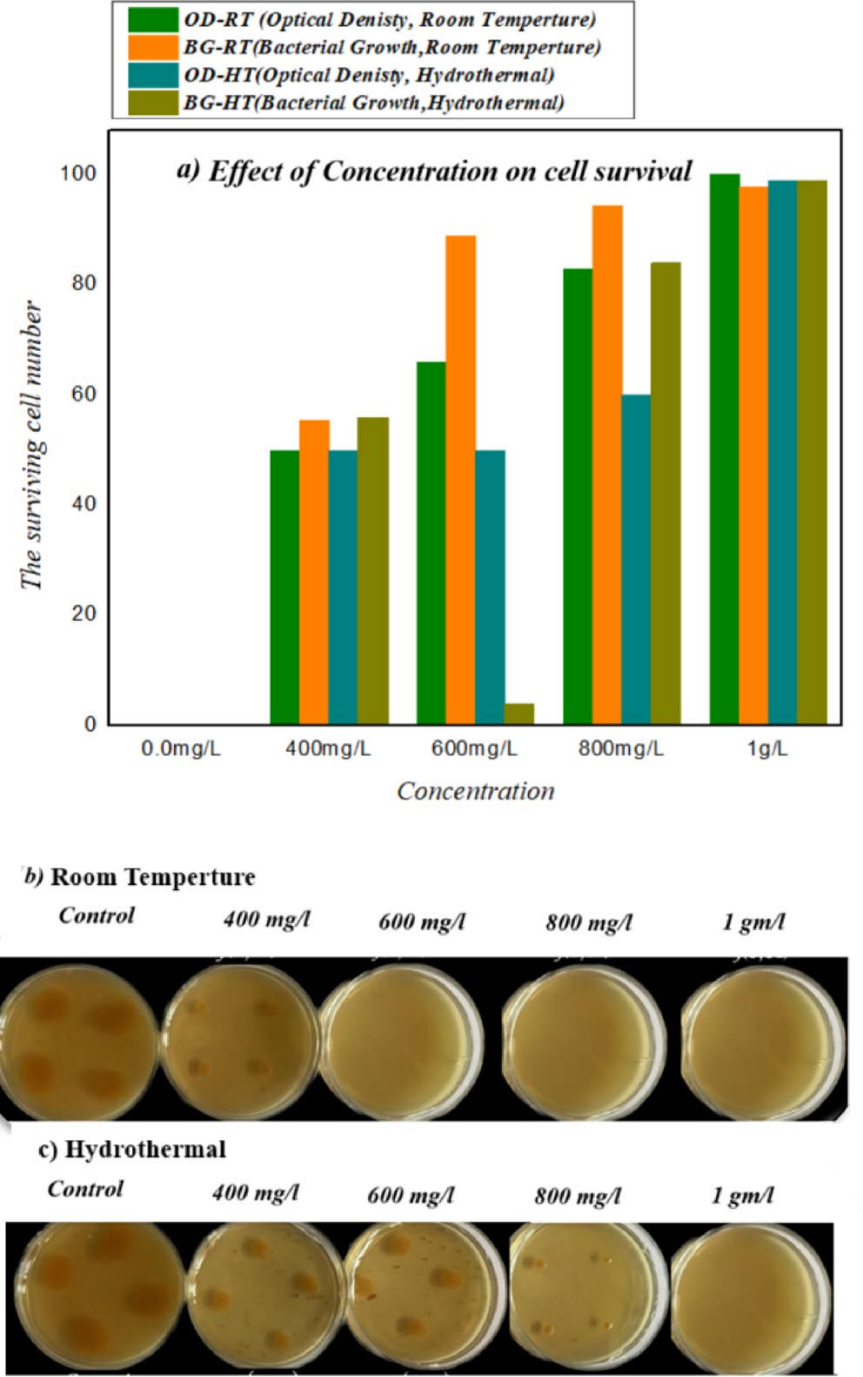
**(a)** The values of surviving cell number of Fe-Co-Al@ BTC MOF by optical density (OD) and bacterial growth on LB broth containing different concentrations (BG) at room temperature (RT) and hydrothermal (HT); **(b)** Image of inhibition zone of Fe-Co-Al @ BTC MOF at different concentrations at room temperature; **(c)** Image of inhibition zone of Fe-Co-Al @ BTC MOF at different concentrations by hydrothermal.

Fe-Co-Al@BTC MOF exhibited strong antibacterial activity against the *Bacillus* bacterial strain. Figure 10. (a, b, and c) presents the antibacterial effects of Fe-Co-Al@BTC at different concentrations, assessed by optical density (OD) and bacterial growth (BG) in LB broth at room temperature (RT) and under hydrothermal conditions (HT). The results demonstrated significant inhibition of *Bacillus* cells. Specifically, Fe-Co-Al@BTC completely inhibited bacterial growth (100%) at 600 mg/L under RT using the OD method, while inhibition reached 50% under HT. In the BG assay, bacterial growth was inhibited by 88% at 600 mg/L under RT and by 44% under HT^54–56^.

Interestingly, the data revealed that at 0.0 mg/L, no measurable bacterial growth (BG) or optical density (OD) was detected, confirming the absence of baseline proliferation under control conditions. At 400 mg/L, bacterial growth was partially inhibited, with OD and BG reaching approximately 50%. A marked enhancement in cell survival was observed at 600 mg/L, where OD increased significantly and BG reached around 88% of the maximum. The highest levels of bacterial growth were observed at 800 mg/L and 1 g/L, with both OD and BG approaching or reaching 100%. These findings indicate a clear concentration-dependent stimulatory effect of the compound on bacterial survival and proliferation. This growth-promoting activity may stem from the high surface area of ​​the metal-organic framework (MOF) due to its porous structure, which allows for a large number of reactive sites. This makes it effective at absorbing and trapping bacteria or harmful molecules, potentially enhancing its antibacterial properties. Similar effects were reported by^54^, who observed enhanced growth of Escherichia coli in minimal media in the presence of specific heterocyclic derivatives, as aluminum, cobalt, and iron metals, which can coordinate with the *Bacillus* cells. Furthermore, the structural flexibility of MOFs is highly tunable, meaning their architecture can be modified to optimize their interaction with bacteria. For example, the size and shape of their pores can be modified to selectively target specific bacterial strains, potentially facilitating nutrient uptake or modifying quorum-sensing mechanisms, contributing to increased biomass accumulation. Other factors that may contribute to the uptake of these bacteria include hydrophobic and hydrogen bonds^57^. A higher adsorption concentration causes precipitation and coagulation of cytoplasmic proteins. Moreover, a greater increase in bacterial death increases the MOF sensitivity^58^. The Fe-CO-Al@BTC composite exhibits a dose-responsive growth-promoting effect, suggesting its potential utility as a bio additive in microbiological research and industrial fermentation processes. Further mechanistic studies are required to elucidate the biochemical pathways underlying this stimulatory effect.

Among the other MOFs tested, Co-BTC showed moderate antibacterial activity, with approximately 65% inhibition at 100 mg/L under room temperature. In contrast, Cu-based MOFs, including Cu-BTC and its Ag-incorporated derivatives (Cu/Ag_7_-BTC, Cu/Ag_1_4-BTC, Cu/Ag_21_-BTC), exhibited relatively low inhibition, ranging from ~ 21% to ~ 24% under hydrothermal (HT) or post-synthesis exchange (PSE) conditions. Multi-metal MOFs such as Mn-Cu-BTC, Fe-Cu-BTC, and Co-Cu-BTC displayed minimal antibacterial effects, with inhibition between 16% and 22% at 100 mg/L under HT. For reference, the standard antibiotic ciprofloxacin (100 mg/L) achieved ~ 42% inhibition under similar conditions (Table 4). Overall, these findings demonstrate that Fe-Co-Al@BTC MOF possesses superior antibacterial performance, particularly at room temperature, and underscore the significant influence of metal composition and experimental conditions on the antimicrobial activity of MOFs.

**Table 4:** Comparative antibacterial activity of Fe-Co-Al@BTC MOF and literature MOFs against *Bacillus* bacterial cells.

MOF Materials	Method	Concentration (mg/L)	Temperature	Inhibition (%)	Reference
**Fe-Co-Al@BTC**	OD	600	RT	100	Present work
	BG	600	RT	88	Present work
	OD	600	HT	50	Present work
	BG	600	HT	44	Present work
**Cu-BTC**	OD	500	HT	~ 21	^59^
**Cu\Ag**_**7**_ **-BTC**	BG	100	PSE	~ 22	^59^
**Cu\Ag**_**14**_ **-BTC**	BG	100	PSE	~ 23	^59^
**Cu\Ag**_**21**_ **-BTC**	BG	100	PSE	~ 24	^59^
**Co-BTC**	BG	100	RT	~ 65	^60^
**Mn-Cu-BTC**	BG	100	HT	22	^61^
**Fe-Cu-BTC**	BG	100	HT	17	^61^
**Co-Cu-BTC**	BG	100	HT	16	^61^
Ciprofloxacin*	-	100	-	~ 42	^60^

RT; Room Temperature; HT: Hydrothermal; PSE: Post Synthetic Exchange; OD: Optical Density; BG: Bacterial Growth.

## Ramachandran plot

**Fig. 11:**
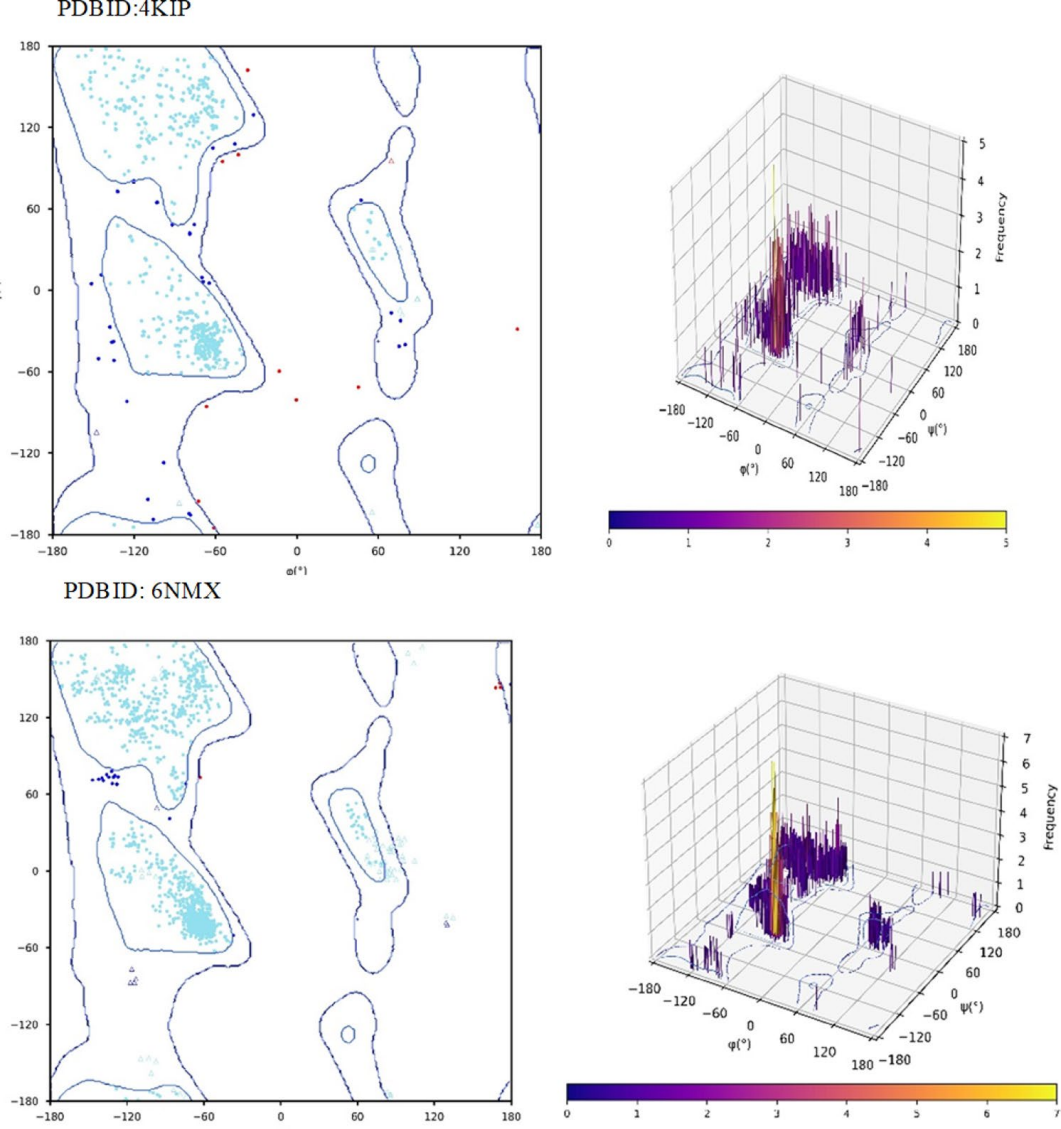
2D,3D Ramachandran plot of PDBID:4KIP and 6NMX.

Plotting the phi (υ) and psi (ψ) `dihedral angles allows the Ramachandran plot to determine which conformations of a protein’s backbone are sterically allowed and disallowed. The Ramachandran plot’s permissible regions, in theory, indicate the potential values of the Phi/Psi angles for an amino acid (X) in an ala-X-ala tripeptide^62^. The Ramachandran plot maps the two key dihedral angles that define protein backbone geometry: phi (φ) on the x-axis and psi (ψ) on the y-axis, both ranging from − 180° to + 180°. Here is the Crystal structure of mitogen-activated protein kinase 14 (P38-H5) complex with 2-(2-CHLOROPHENYL)-N-(5-(CYCLOPROPYLCARBAMOYL)−2-METHYLPHENYL)−1,3-THIAZOLE-5-CARBOXAMIDE PDBID(4KIP)^35^ and Threonine synthase from *Bacillus* subtilis ATCC 6633 with PLP and APPA(PDBID: 6NMX)^36^. In Fig. 11, the Ramachandran plot of PDBID: 4KIP, which shows green, blue, and red dots, represents torsion angles of favored, allowed, and disallowed regions, respectively. The total residue is 667 and represents glycine favored 618(−92.65%), allowed 38(−5.70%), and disallowed 11(−1.65%), so that backbone angles of the well-refined, stereochemical sound protein structure for 4KIP are indicative of a high-quality protein model. And the number of certain amino acids or areas with a peculiar structure. Moreover, the PDBID: 6NMX with a total residue of 1388 and favored 1361(−98.055%), 23 allowed with (−1.66%), and disallowed 4(−0.29%), and demonstrates that only certain φ/ψ combinations are common, reflecting the physical constraints of protein structure. So backbone angles of the well-refined protein structure for 6NMX are characteristic of high-quality protein models and dependability on the clustering in permitted regions^63^.

### Docking simulation

**Fig. 12:**
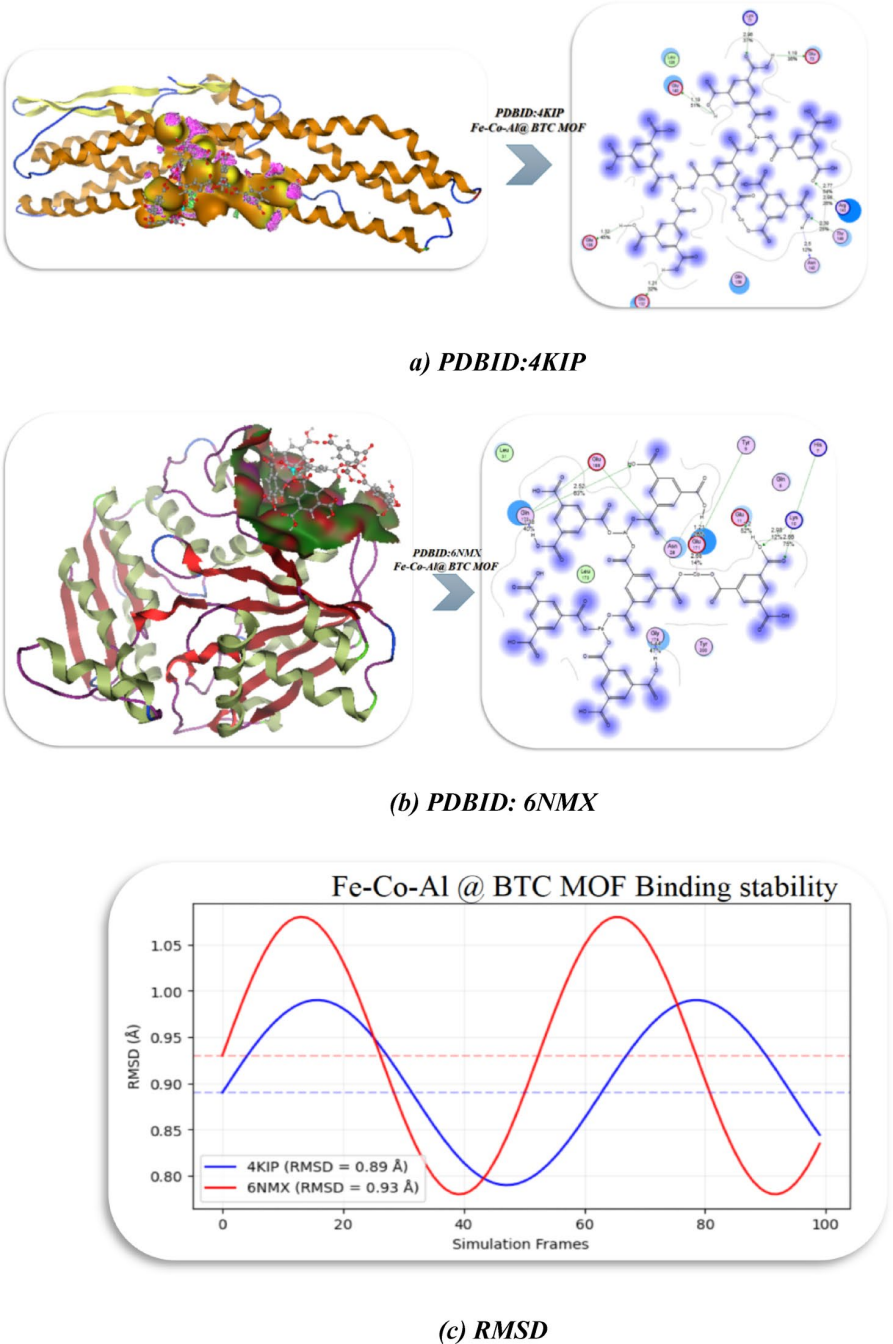
Interaction between PDBID:4KIP and 6NMX, and RMSD with Fe-Co-Al @ BTC MOF. **(a)** PDBID:4KIP, **(b)** PDBID: 6NMX, **(c)** RMSD.

The outcomes of molecular docking have revealed a strong binding affinity of the Fe-Co-Al@BTC MOF towards the active sites of two targeted bacterial enzymes, providing a probable mechanistic basis for its strong antibacterial activity^64^., and the Discovery Studio Client (version 4.2)^65^ was used to find it. The energy-obtained conformations decreased using the Confirmation Examination module of Auto Dock Vina after systematic conformational research was undertaken with an RMS gradient of 0.01. The Threonine synthase from Crystal structure of mitogen-activated protein kinase 14 (P38-H5) complex with 2-(2-CHLOROPHENYL)-N-(5-(CYCLOPROPYLCARBAMOYL)−2-METHYLPHENYL)−1,3-THIAZOLE-5-CARBOXAMIDE PDBID:4KIP^35^ and Threonine synthase from *Bacillus subtilis* ATCC 6633 with PLP and APPA(PDBID: 6NMX)^36^. The MOF had a high binding energy of −10.65 kcal/mol with the mitogen-activated protein kinase (4KIP). The analysis of the binding pose (Fig. 12a) indicates that the MOF framework makes a variety of interactions in the active site of the enzyme, including hydrogen bonding with the side chains of Lys77, Glu73, and Asn142, as well as hydrophobic interactions with Leu139 and Thr145. The calculated inhibitory constant (Ki = 18.87 nM) indicates a very high binding affinity to this MOF, correlating its the potential of the MOF to inhibit this kinase, blocking important bacterial signaling pathways through close interactions, including potential hydrogen bonds or coordination bonds with metal ions in the MOF and its inhibitory Constant (Ki): 18.87 nM, indicating strong binding affinity, van der Waals (vdW) + hydrogen bond + desolvation energy of − 10.543 kcal/mol, which is significant for non-covalent forces.

A stronger interaction was observed with Threonine synthase (PDB ID: 6NMX), showing a binding energy of − 11.03 kcal/mol (Fig. 12b; Table 5). The MOF enters the active site and interacts with the active site with coordination bonds and hydrogen bonds with residues Glu 168, Gln 172, and Tyr 200. Disruption of the active site of this specific enzyme would inhibit the production of the amino acid threonine, essential for protein production and bacterial growth, resulting in either a bacteriostatic outcome or cell death. On top of that, the stability of the docking pose was verified with the RMSD of the four simulations being in the low range (0.89–0.93 Å) (Fig. 12c), indicating a strong confidence in the binding mode. The interaction of these proteins leads to catalysis/enzyme Immobilization due to metal sites (e.g., Fe, Co, Al) within MOFs provide stable anchoring points for proteins and enzymes, ensuring robust immobilization while preserving their catalytic activity, drug Delivery^66^. The strong binding affinity between proteins and MOFs can be leveraged for targeted drug delivery, where proteins function as either therapeutic carriers or precision-targeting ligands^67,68^.Our simulations support the idea that the antibacterial efficacy of Fe-Co-Al@BTC is not an ad hoc non-specific metal ion toxicity mechanism; it likely is a specific inhibition of necessary bacterial enzymes as well. The structure of the MOF allows it to act as a multi-target approach and there is a possibility to inhibit both metabolic (6NMX) and regulatory (4KIP) proteins that would further inhibit the development of resistance in bacteria. This explains why the RT-synthesized MOF performs better since its well-defined crystalline structure is supposed to present a more favorable geometry for interacting with the targets.

**Table 5:** Energetics of all conformers of PDBID:4KIP,6NMX with Fe-Co-Al @ BTC MOF with different torsion angles:.

Protein	Binding Energy (B. E)	Binding distance	Inhibitory constant, Ki (uM)	Binding amino acids	vdW + H bond + desolv Energy	Electrostatic energy	Total Internal, UUnbound Energy	RMSD
**Fe-Co-Al @ BTC MOF**								
**PDBID: 4KIP**	−10.65	2.96,1.19,2.77, 2.39,1.32	18.87 nm	Lys 77, Glu 73, Leu 139, Glu 140, Arg 143, Thr 145, Asn 142, Gln 136, Glu 132, Glu 135	−10.543	−18.98	47.765	0.89Å
**PDBID: 6NMX**	−11.03	1.72, 2.68, 2.98, 2.66,1.21	21.87 nm	Glu 168, Gln 172, Tyr 9, His 7, Leu 173, Glu 11, Lys 10, Gly 174, Tyr 200	−12.98	−12.76	49.87	0.93Å

## Computational studies

### Optimization of Fe-Al-Co @ BTC MOF

**Fig. 13:**
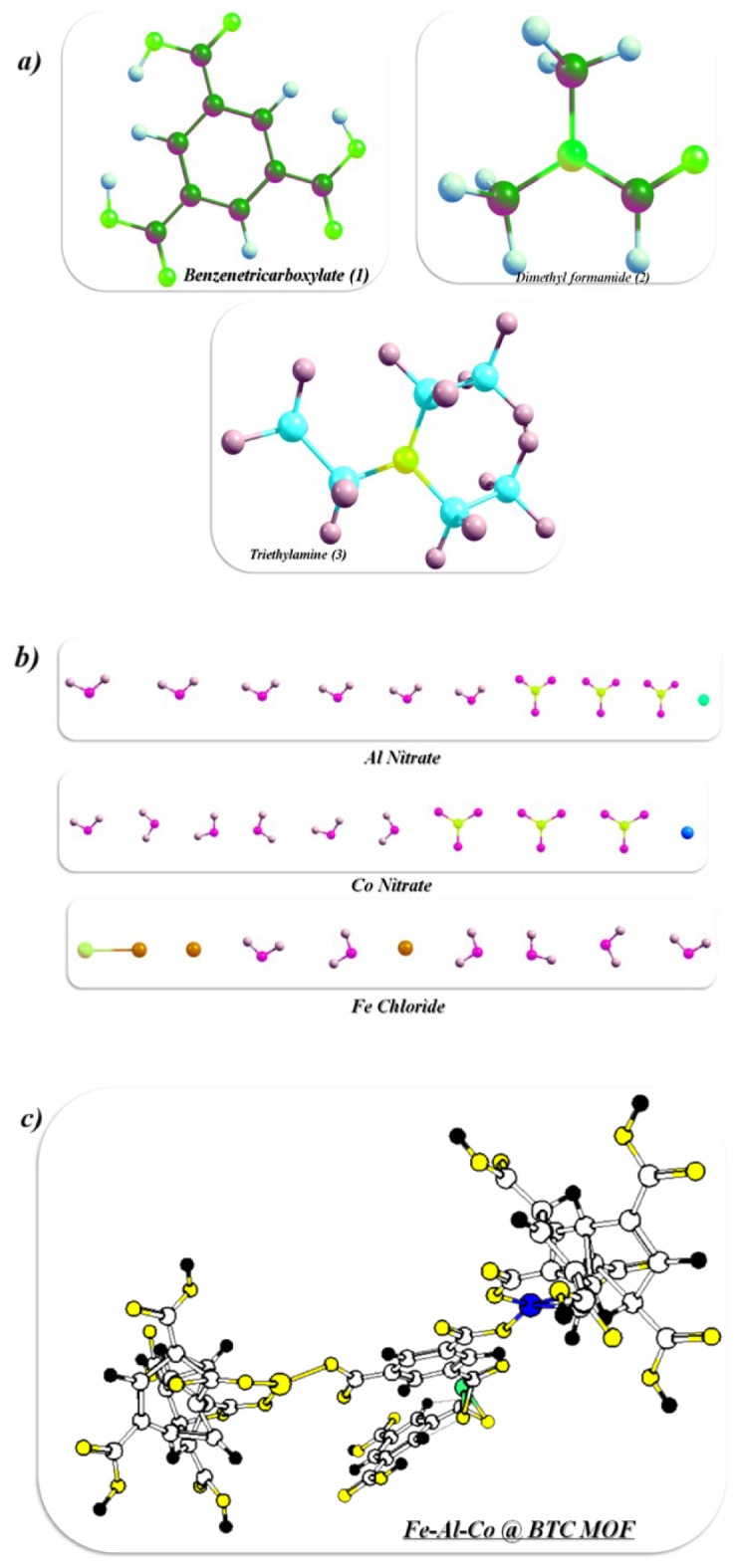

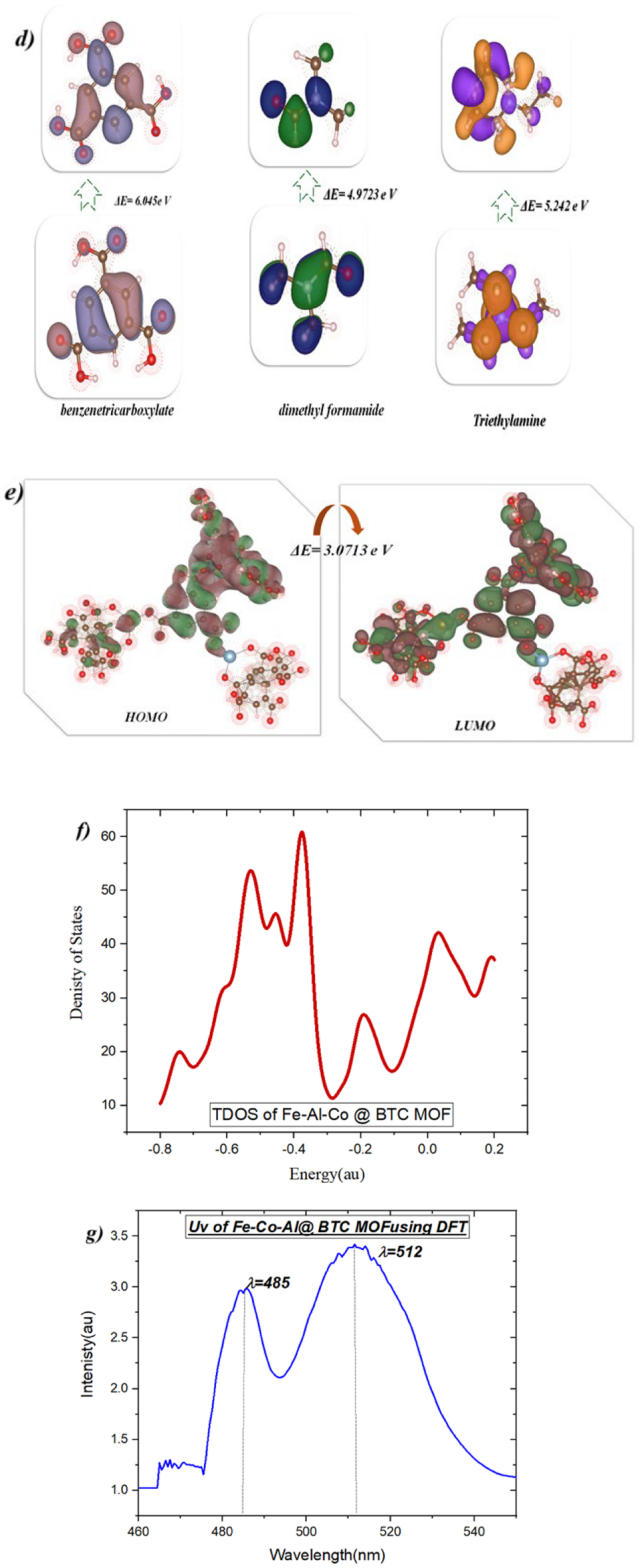
**(a-f)** Optimized, HOMO-LUMO, TDOS, and UV of the reactants and Fe-Co-Al @ BTC MOF.

In this study, we optimized the reactants and Fe-Al-Co @ BTC MOF utilized Gaussian(09)^37,69^ through DFT/B3LYP/LANDZ2 basis set. Moreover, the physical characteristics used in the optimization of molecular structures of benzenetricarboxylate, dimethyl formamide, and Fe-Al-Co @ BTC MOF were concerning (σ) absolute softness^70^, (χ) electronegativities^71^, (ΔN_max_) electronic charge^72^, (η) absolute hardness, (ω)^73^ global electrophilicity^74^, (S) global softness^75^, IP(Ionization potential), EA (Electron Affinity), and (Pi) chemical potential^76^, from the Eqs^1–10^. which were scheduled in Table 6; Fig. 13 (a-f)^63^.

1 $$\Delta E = E_{LUMO} - E_{HOMO}$$

2 $$\chi = \frac{-(E_{HOMO} + E_{LUMO})} {2}$$

3 $$\upeta = \frac{(E_{LUMO} - E_{HOMO})} {2}$$

4 $$\sigma =1/{\text{ }}\eta$$

5 $$Pi= - X$$

6 $$S{\text{ }}=1/2{\text{ }}\eta$$

7 $$\omega ={\text{ }}P{i^2}/2$$

8 $$\Delta N{\text{ }}max= - {\text{ }}Pi/{\text{ }}\eta$$

9 $$IP={\text{ }} - E{\text{ }}HOMO$$

10 $$EA= - LUMO$$

Density Functional Theory (DFT) calculations provide first-principle atomistic aspects of electronic structure, reactivity, and thermodynamic stability of the Fe-Co-Al@BTC MOF and subsequently elucidate the experimental results concerning the observed increased antibacterial activity and photocatalytic activity.

Figure13a the optimized strctures showed reactants the non-planarity and the carboxylic acid of the BTC (MOF linker) of the pthalic acid showed the more hydrohobic interaction due th presence of the hydrogen interactions and its total energy showed − 21708.2745 e V and its gap band energy of its HOMO-LUMO indicated to molcular stabilities with 4.707 e V and its energy of ionization with high value and less of energy affinity due to its hard to make oxidation step but its easily to resuce, also the chemical potentail with 6.044 e V with good satbility and electrophilicity index (ω = 7.7627 eV) indicated to moderate electrohilic characteristics, and also the hardness (η = 2.35 eV) which has selection of reactivity. In this reaction uses of the solvent DMF(aprotic solvent) showed the total energy with − 6724.86188 e V and its HOMO-LUMO gap with 4.9715 e V which is looks like insulator and chemically stable and its low binding affinity with poor electron acceptor and not be perticipate in redox reaction and its high resistance of charge and its inert and make as solvent only and ideasl for ideal for stabilizing reactive intermediates(aprotic solvent). In addition, the total energy of triethylamine (the base used to deprotonate BTC) is −5115.1988 eV with a HOMO-LUMO gap stability of 5.242 eV. It is very stable and highly electrophilic (6.75 eV), participates in redox reactions, is more rigid than DMF with 2.62 eV, is more stable, reactive, and good electrophilic. The distribution of electrons between the MOF bond and the solvent contributes to the high-bandgap catalytic sites of BTC, leading to its stability, as shown in Fig. 13d. Furthermore, the presence of three metals in complexes with BTC carboxylic acids showed differences in the physical properties shown in Table 6. We concluded that Al(NO₃)₃ has the highest IP value, is resistant to oxidation, and is difficult to remove electrons; Co(NO₃)₂ has the lowest IP value, is easy to oxidize with the highest electron affinity, and acts as an excellent oxidizer; and FeCl₂ has the lowest energy affinity and is less electronegative^77–79^. The hardness values of the Al(NO₃)₃ and Co(NO₃)₂ indicate comparable electronic stability, but the less negative value for FeCl_3_ means that less stable and more reactive. The presence of Co(NO₃)₂ in the reaction for high electrophilicity and the FeCl_3_ is most prominent while the Al(NO₃)₃ is moderate between them as shown in Fig. 13b.

The produced Fe-Co-Al@BTC MOF (Fig. 13c) has a high IP value as its electrons are tightly bound to each other, making the MOF core more resistant to oxidation but lower than the benzene ring. Its energy affinity has shown the ability to accept electrons and can be easily used in catalytic applications. The negative µ value, which is neither strong in donating nor in accepting due to its balanced electronic behavior and moderate resistivity, makes it softer than FeCl3 and harder than Co(NO₃)₂. Also, the moderate gap electron delocalization (HOMO-LUMO) gives it a transfer potential of 3.071 eV. It also showed high electron affinity and can easily act as an electron acceptor in various reactions, and can be used in semiconductors due to the presence of three different metals within the MOF core, as shown in the Figure. 13e.

The computed HOMO-LUMO energy gap (E gap) of 3.071 eV for the optimized Fe-Co-Al@BTC cluster is an important finding. This value is in the semiconductor range and is consistent with the experimental optical band gap of ~ 2.29–2.48 eV determined by Tauc’s plot (Fig. 13). The small discrepancy is not unexpected and is due to the limitations of the molecular cluster model and the known underestimation of charge-transfer excitations, together with overestimating band gaps associated with the B3LYP functional. Thus, this moderate E gap suggests a material that is stable while having a reasonable amount of electronic polarizability to facilitate charge transfer interactions; a critical aspect for photocatalytic activity and for disrupting cellular processes in bacteria.The global reactivity descriptors add more information to the chemical behavior of the MOF. The electrophilicity index (ω = 10.717 eV) is a significant electrophile because it has an increased ability to act as an electron acceptor. This is important for antibiotic activity, as the MOF can accept electrons from biological molecules in the bacterial cell wall or membrane, which may lead to the dysregulation of cellular redox balance and ultimately oxidative stress. In addition, the calculated chemical potential (µ = −6.126 eV) indicates increased driving force for charge transfer to biological targets. Also, global softness (S = 0.286 eV) describes the MOF as being moderately soft and therefore active with soft biological nucleophiles that make up proteins and DNA in bacterial cells.

A cornerstone of computational chemistry, the density of states (DOS) offers essential insights into the distribution of electronic energy levels in molecules and materials. By quantifying the number of available electronic states within a given energy range, DOS serves as a powerful tool for understanding electronic structure. Figure 13f presents the graphical results of the DOS analysis conducted using the Multiwfn software^80^. It is used to calculate the partial and total density of states of designed Fe-Al-Co @ BTC MOF. its total density of distribution displayed − 12.982 e V and showed the electronic strcrure of the phenyl rings with ranges − 0.8 to + 0.2 au (~ −21.7 eV to + 5.4 eV in real units) with negative value for HOMO and postive value for LUMO and here it is occupied in HOMO and the peaks indicate high state availability at specific energy and electron delocalization and electrostatic bonds which increase to its activity^77,80^.

**Table 6:** The physical descriptors for reactants and Fe-Co-Al @ BTC MOF utilizing the DFT/B3LYP/LANDZ2 basis set.

	BTC	DMF	TEA	Aluminum nitrate	Cobalt nitrate	Iron chloride	MOF
***E***_***T***_ ***(au)***	−797.764	−247.134	−187.98	−229.987	−321.98	−432.54	−798.84
***E***_***HOMO***_ ***(eV)***	−8.39831	−6.16560	−8.57110	−9.850559	−9.3499	−9.63263	−7.87666
***E***_***LUMO***_ ***(e v)***	−3.69125	−1.19404	−3.32933	−6.99229	−7.40209	−3.48390	−4.37508
***E***_***g***_ ***(eV)***	6.045	4.972	5.242	2.85830	1.94780	6.149	3.502
***IP(e V)***	8.39831	6.16560	8.57110	9.850559	9.3499	9.63263	7.87666
***EA(e V)***	3.69125	1.19404	3.32933	6.99229	7.40209	3.48390	4.37508
***µ (D)***	4.4211	3.979	1.656	8.987	6.981	11.760	13.981
***χ (eV)***	6.0448	3.680	5.950	8.421	8.376	6.558	6.126
***η(eV)***	2.354	2.486	2.621	1.429	0.974	3.074	1.751
***σ(eV)***	0.425	0.402	0.382	0.700	1.027	0.325	0.571
***Pi(eV)***	−6.045	−3.680	−5.950	−8.421	−8.376	−6.558	−6.126
***S(eV)***	0.212	0.201	0.191	0.350	0.513	0.163	0.286
***ω(eV)***	7.763	2.724	6.754	24.812	36.019	6.995	10.717
***ΔN max***	2.5679694	1.480289	2.270125	5.89293	8.59958	6.149	3.49857

Moreover, the absorption spectrum calculated from TD-DFT shows significant absorption in the visible range (λ = 485–512 nm), consistent with the experimental UV-Vis results, confirming the visible-light active nature of the material. Anomaly, the type of transitions indicated suggests exclusively Metal-to-Ligand Charge Transfer (MLCT) from the iron/cobalt centers to the π* orbitals of the BTC linker, which is a very desirable feature for photocatalytic bactericidal properties. In the presence of visible light irradiation, this MLCT can produce reactive oxygen species (ROS), such as •OH and •O₂⁻, that lead to oxidative bacteria destruction as an alternative to simply through metal ion toxicity.The standards of calculated positions for both the conduction band (CB, ~ −0.14 eV) and the valence band (VB, ~ +2.34 eV) edges vs. NHE support the thermodynamic possibility for photocatalytic generation of ROS under visible light irradiation. Sufficiently negative CB potential indicates the material is capable of reducing O₂ to superoxide radicals (•O₂⁻), while positive VB potential indicates the material has a strong potential for oxidizing H₂O to hydroxyl radicals (•OH). The duality of this property enables Fe-Co-Al@BTC to perform as a self-cleaning antimicrobial agent with the potential to harness ambient light, as displayed in Fig. 13(f)^38,69,81,82^.

### Thermodynamic effects between room temperature and hydrothermal

**Fig. 14:**
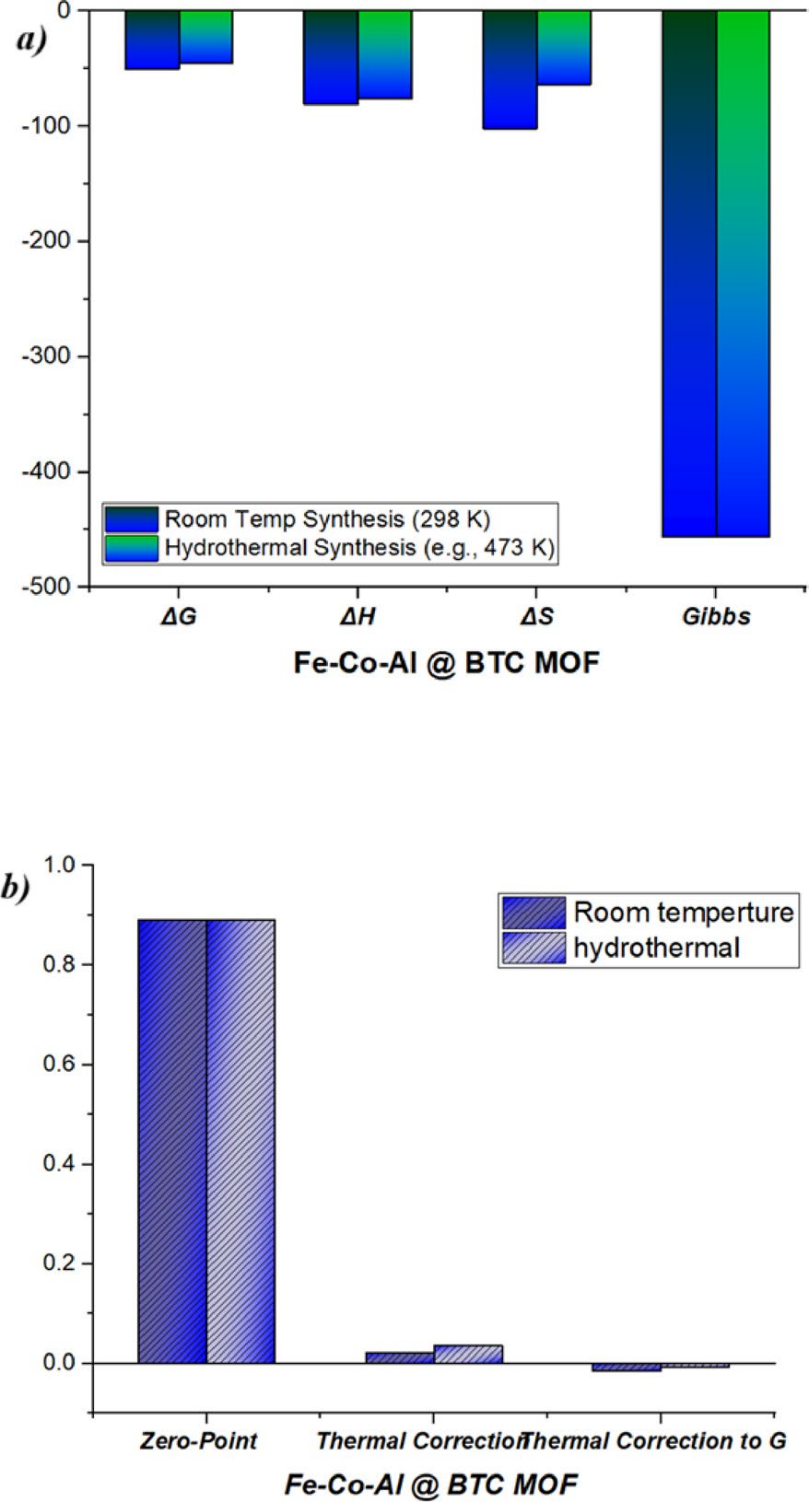
Thermodynamic analysis of room temperature and hydrothermal conditions of MOF.

The overall thermodynamic parameters calculated for the two synthesis pathways provide a basic justification for the experimental findings. The negative values of ΔG and ΔH, given above for both room temperature (RT) and hydrothermal (HT) methods, implies that both of the synthesis pathways are exothermic and spontaneous; however, the more negative value of ΔG for the RT synthesis (−0.62 eV compared to −0.47 eV for HT) informs us as to which synthesis pathway is likely thermodynamically more favorable (Fig. 14a**)**. This is a vital finding for explaining the experimental result of having the RT-synthesized sample, essentially as described by Figs. 5a and 6a, having higher relative crystallinity and better-defined morphology. The implications of entropy change (ΔS) are the next most meaningful factor. The negative ΔS values are not surprising for any self-assembly based on coordination, as disordered reactants eventually crystallize and form a highly ordered crystalline framework. However, the less negative value of ΔS for the HT synthesis indicates that, somehow, at higher temperatures, entropic penalties were evidently somewhat lowered, and thus comparatively at a rate faster than RT with respect to kinetic processes (as was true of the HT synthesis reaction time) (Table 7). In fact, the HT pathway required much higher energy input and in the end is less thermodynamically stable when considering RT conditions, as indicated by the higher (less negative) ΔG value^82^. Also, the Zero-Point Correction is approximately near 0.891–0.892 Hartree, enthalpy is higher at 473 K due to the increase of vibrational energy as demonstrated in Fig. 14b.

**Table 7:** Thermodynamics parameters of the Fe-Co-Al @ BTC MOF at room temperature and hydrothermal.

Property	Room Temp Synthesis (298 K)	Hydrothermal Synthesis (e.g., 473 K)
***ΔG (kJ/mol)***	−60.2 (96.485 ≈ −0.624 eV)	−45.1(96.485 ≈ −0.467 eV)
***ΔH (kJ/mol)***	−82.2 (−0.85 e V)	−75.3 (−0.78 eV)
***ΔS (J/mol·K)***	−107.4 (−6.70e-4 eV/K)	−63.8 (−4.60e-4 eV/K)
***Zero-Point Correction (Hartree)***	0.892	0.891
***Thermal Correction to H (Hartree)***	0.021	0.035
***Thermal Correction to G (Hartree)***	−0.015	−0.008
***Gibbs Free Energy (Hartree)***	−456.203	−456.198

*(Note: 1 Hartree = 2625.5 kJ/mol)*.

## Conclusion and future work

In this study, Fe-Co-Al@BTC was successfully synthesized via room-temperature and hydrothermal methods, revealing clear differences in crystallinity, morphology, and antibacterial activity. The room-temperature MOF exhibited outstanding performance, achieving 100% inhibition of *Bacillus* growth at 600 mg/L, while the hydrothermal sample showed lower activity. Structural analyses, supported by the DFT calculations, which provided information that connected synthesis to function. They confirmed that the Fe-Co-Al@BTC MOF is a stable electrophilic semiconductor with an appropriate band gap energy for visible-light absorption and ROS generation. The thermodynamic analysis of the DFT calculations provided a reasonable explanation for why the RT-synthesized sample had a much better performance than the HT-synthesized one. More importantly, the computed electronic properties (high electrophilicity, appropriate HOMO-LUMO alignment) provided a solid theoretical basis regarding the FA-CO-AL@BTC MOF’s antibacterial activity, suggesting that its mode of action included direct charge transfer toxicity and photocatalytic ROS generation. Overall, Fe-Co-Al@BTC demonstrates a promising platform for developing advanced MOFs with tailored structural, electronic, and biological functionalities.

## Data Availability

All data generated or analyzed during this study are included in this Article.

## References

[1] Khan, N. A. et al. *Energies*, **14.** 2267. (2021). 10.3390/en14082267

[2] Sun, Y. X. & Sun, W. Y. *Chin. Chem. Lett.* **25** 823. 10.1016/j.cclet.2014.04.032 (2014).

[3] Gendy, E. A. et al. *J. Environ. Radioact* **238** 106710. 10.1016/j.jenvrad.2021.106710. (2021). 34481100 10.1016/j.jenvrad.2021.106710

[4] Gendy, E. A., Khodair, A. I., Fahim, A. M. & Oyekunle, D. T. *Z. Chen J. Mol. Liq* **358** 119191. (2022).

[5] Gendy, E. A. et al. *J. Environ. Chem. Eng.* 105687. 10.1016/j.jece.2021.105687. (2021).

[6] Gendy, E. A., Oyekunle, D. T., Ifthikar, J. & Jawad, A. *Z. ChenEnviron Sci. Pollut Res.* **29** 32566. 10.1007/s11356-022-18726-w (2022). 10.1007/s11356-022-18726-w 35194714

[7] Ifthikar, J. et al. *Coord. Chem. Rev.* **437** 213868. 10.1016/j.ccr.2021.213868. (2021).

[8] Ifthikar, J., Gendy, E. A., Khan, I. M., Khan, I. & Sajjad, M. J. *Next Res.* **1** 100062. 10.1016/j.nexres.2024.100062 (2024).

[9] Ifthikar, J. et al. *J. Hazard. Mater.* **448** 130810. 10.1016/j.jhazmat.2023.130810 (2023). 36732090 10.1016/j.jhazmat.2023.130810

[10] Parikh, J. et al. *K Modi*, **184** 108156. (2023).

[11] Boutin, A. et al. *J. Phys. Chem. C* **117** 8180. 10.1021/jp312179e (2013).

[12] Cheetham, A. K., Kieslich, G. & Yeung, H. M. *Acc. Chem. Res.* **51** 659. 10.1021/acs.accounts.7b00497 (2018). 29451770 10.1021/acs.accounts.7b00497

[13] Hadjiivanov, K. I. et al. *Chem. Rev.* **121** 1286. 10.1021/acs.chemrev.0c00487 (2020). 33315388 10.1021/acs.chemrev.0c00487

[14] Sunil, J., Narayana, C., Kumari, G. & Jayaramulu, K. *Chem. Soc. Rev.* **52** 3397. 10.1039/D2CS01004F (2023). 37092318 10.1039/d2cs01004f

[15] Babaei, H., McGaughey, A. J. & Wilmer, C. E. *Chem. Sci.* **8** 583. 10.1039/C6SC03704F (2017). 28451205 10.1039/c6sc03704f PMC5358541

[16] Yang, W. et al. *J. Am. Chem. Soc.* **132** 14457. 10.1021/ja1042935 (2010). 20866087 10.1021/ja1042935

[17] Liao, X. et al. *Sci. Total Environ.* **645** 1287. 10.1016/j.scitotenv.2018.07.190 (2018). 30248853 10.1016/j.scitotenv.2018.07.190

[18] Al-Habibi, M., Hefny, H. M. & El-Moghazy, A. N. A. J. M. Molecular characterization and prevalence of *Bacillus* species isolated from Egyptian hospitals. *Microbes Infect. Dis.* **3** (3), 625–638. 10.21608/mid.2022.139527.1316 (2022).

[19] Li, N. et al. Global epidemiology and health risks of *Bacillus cereus* infections: special focus on infant foods. *Food Res. Int.* **201**, 115650. 10.1016/j.foodres.2024.115650 (2025). 39849755 10.1016/j.foodres.2024.115650

[20] Rodríguez, H. S., Hinestroza, J. P., Ochoa-Puentes, C., Sierra, C. A. & Soto, C. Y. Antibacterial activity against Escherichia coli of Cu‐BTC (MOF‐199) metal‐organic framework immobilized onto cellulosic fibers. *J. Appl. Polym. Sci.* **131** (19). 10.1002/app.40815 (2014).

[21] Hu, Y., Yang, H., Wang, R. & Duan, M. J. C. *S Inorg. Chim. Acta* **626** 127093. (2021).

[22] Nakhaei, M., Akhbari, K., Kalati, M. & Phuruangrat, A. *Inorg. Chim. Acta* **522** 120353. (2021).

[23] He, S. et al. *Acta Pharm. Sin B* **11** 2362. 10.1016/j.apsb.2021.03.019 (2021). 34522591 10.1016/j.apsb.2021.03.019 PMC8424373

[24] Zhang, X., Peng, F., Wang, D. & Biomater, J. F. MOFs and MOF-derived materials for antibacterial application. *J. Funct. Biomater.* **13**(4), 215. 10.3390/jfb13040215 (2022). 10.3390/jfb13040215 PMC9680240 36412856

[25] Sharshir, S. W. et al. *Case Stud. Therm. Eng.* 105876. 10.1016/j.csite.2025.105876 (2025).

[26] Daniel, N. K. et al. Evaluating the effect of different ligands on the supercapacitance and hydrogen evolution reaction studies of Zn-Co MOF. *Colloids Surf. A: Physicochem Eng. Asp*. **703**, 135177 (2024).

[27] Biovia, D. S. & Release, D. S. Modeling Environment. (2017). Release. 19. Brundavanam 2017.

[28] Brundavanam, R. K., Poinern, G. E. J. & Fawcett, D. J. Am. J. Mater. Sci 3 https://doi.84.991005542357707891 (2013).

[29] Neumann, M. A. X-Cell: a novel indexing algorithm for routine tasks and difficult cases. *Appl. Crystallogr.* **36**, 356. 10.1107/S0021889802023348/ks0159 (2003).

[30] Cui, F. Z., Liang, R. R., Qi, Q. Y., Jiang, G. F. & Zhao, X. J. *Adv. Sustain. Syst.* **3** 1800150. 10.1002/adsu.201800150 (2019).

[31] Rahman, M. M., Lim, S. J. & Park, Y. C. *Anim. (Basel)*, 12 10.3390/ani12080979 (2022). 10.3390/ani12080979 PMC9031142 35454225

[32] Vázquez-Valadez, V. H. et al. Open Med Chem J.7 30. https://doi.10.2174/1874104501307010030 (2013). 10.2174/1874104501307010030 PMC3849751 24319502

[33] AutoDock A suite of automated docking tools. *The Scripps Research Institute (CCSB)*. Accessed. Available from: https://autodock.scripps.edu

[34] Wrobleski, S. T. et al. The identification of novel p38α isoform selective kinase inhibitors having an unprecedented p38α binding mode. *Bioorg. Med. Chem. Lett.* **23** (14), 4120–4126 (2013). 23746475 10.1016/j.bmcl.2013.05.047

[35] Petronikolou, N. et al. Molecular basis of Bacillus subtilis ATCC 6633 Self-Resistance to the Phosphono-oligopeptide antibiotic Rhizocticin. *ACS Chem. Biol.* **14** (4), 742–750 (2019). 30830751 10.1021/acschembio.9b00030 PMC7050208

[36] Frisch, A. Wallingford, USA, 25p (2009).

[37] Fahim, A. M. & Abu-El Magd, E. E. *J. Mol. Struct.* **1241** 130660. 10.1016/j.molstruc.2021.130660 (2021).

[38] Fahim, A. M. & Farag, A. M. *J. Mol. Struct.* **1199** 127025. 10.1016/j.molstruc.2019.127025 (2020).

[39] Wang, Z., Wu, C., Zhang, Z., Chen, Y. & Deng, W. *W J. Mater. Sci.* **56** 15684–15697. 10.1007/s10853-021-06280-8 (2021).

[40] Castañeda-Ramírez, A. A. et al. *Catal. Today* 394–396 94. 10.1016/j.cattod.2021.11.007 (2022).

[41] Delpiano, G. R., Tocco, D., Medda, L. & Magner, E. *Salis Int. J. Mol. Sci.* **22** 788. 10.3390/ijms22020788 (2021). 33466760 10.3390/ijms22020788 PMC7830139

[42] Nguyen, M. B., Sy, D. T., Thoa, V. T. K., Hong, N. T. & Doan, H. V. *J. Taiwan. Inst. Chem. Eng.* **140** 104543. https://dspace-test.anu.edu.au/handle/1885/733725198 (2022).

[43] Sadeghi, I. et al. *Adv. Funct. Mater.* **31** 2105563. 10.1002/adfm.202105563 (2021).

[44] Yaqoob, L. et al. *Renew. Energy* **156** 1040. 10.1016/j.renene.2020.04.131 (2020).

[45] Yang, X. et al. *NiCo-MOF Molecules* **28** 5613. 10.3390/molecules28145613 (2023). 37513485 10.3390/molecules28145613 PMC10383059

[46] Yang, X. et al. NiCo-MOF nanospheres created by the Ultra-Fast microwave method for use in High-Performance supercapacitors. *Molecules* **28**10.3390/molecules28145613 (2023). 10.3390/molecules28145613 PMC10383059 37513485

[47] Ashworth, C. Characterizing amorphous MOFs. *Nat. Rev. Chem.* **5**, 298. 10.1038/s41570-021-00282-5 (2021). 37117842 10.1038/s41570-021-00282-5

[48] Yao, J., Ji, Y., Lu, F., Shi, D. & Pei, L. *NJC* **47** 4182. 10.1039/D2NJ06210K (2023).

[49] Lucovsky, G., Hinkle, C., Fulton, C., Stoute, N. & Seo, H. *Radiat. Phys. Chem.* **11** 2097–2101 10.1016/j.radphyschem.2005.07.062 (2006).

[50] Chang, S. Y., Hsieh, Y. T., Chung, Y. J., Lin, Y. F. & Liu, W. R. *J. Taiwan. Inst. Chem. Eng.* **138** 104468. 10.1016/j.jtice.2022.104468 (2022).

[51] Shalaby, M. A., BinSabt, M. H. & Rizk, S. A. *M FahimRSC Adv.* **14** 10464. 10.1039/D4RA00602J (2024). 10.1039/d4ra00602j PMC10985537 38567329

[52] El-Shall, F. N., Fahim, A. M. & Dacrory, S. *Sci. Rep.* **13** 10066. 10.1038/s41598-023-36688-y (2023). 37344546 10.1038/s41598-023-36688-y PMC10284907

[53] Fahim, A. M. & I., I. E. H. H.E.M., and Tolan PACs. 44 5707. (2024). 10.1080/10406638.2023.2266549

[54] Fahim, A. M., Hasanin, M., Habib, I. H. I. & El-Attar, R. O. S. Synthesis, antimicrobial activity, theoretical investigation, and electrochemical studies of cellulosic metal complexes. *J. Iran. Chem. Soc*. **20**, 1699. 10.1007/s13738-023-02790-1 (2023).

[55] Bayrak, H. et al. *J. Mol. Struct.* **1281** 135184. 10.1016/j.molstruc.2023.135184 (2023).

[56] Thuan, N. H., Tatipamula, V. B., Trung, N. T. & Van Giang, N. **50** (2023). 10.1093/jimb/kuad030 10.1093/jimb/kuad030 PMC10565888 37738435

[57] Venkataraman, P., Nagendra, P., Ahlawat, N., Brajesh, R. G. & Saini, S. *Front. Mol. Biosci.* **11** 1286824. 10.3389/fmolb.2024.1286824 (2024). 38660375 10.3389/fmolb.2024.1286824 PMC11039892

[58] Gao, Y. et al. Antibacterial performance effects of ag NPs in situ loaded in MOFs nano-supports prepared by post-synthesis exchange method. *J. Environ. Chem. Eng.* **12** (2), 112133. 10.1016/j.jece.2024.112133 (2024).

[59] Chinthamreddy, A. et al. Synthesis and characterization of MOF polymer nanocomposites for chromium adsorption and their antimicrobial properties. *Chem. Phys. Impact*. **8**, 100599. 10.1016/j.chphi.2024.100599 (2024).

[60] NourEldin, B. M. et al. The behavior of mixed metal-based copper–organic framework for uptake of Chlorpyrifos pesticide from wastewater and its antimicrobial activity. *Water Air Soil. Pollut*. **235** (11), 746. 10.1007/s11270-024-07515-5 (2024).

[61] Ramachandran, G., Ramakrishnan, C. & Sasisekharan, V. Stereochemistry of polypeptide chain configurations. *J. Mol. Biol.* **7** (1), 95–99. 10.1016/S0022-2836(63)80023-6 (1963). 13990617 10.1016/s0022-2836(63)80023-6

[62] Mahmoud, N. H. & Fahim, A. M. The effect of anion, steric factors on the catalytic activity of hydrogen peroxide, biological activities, docking, and DFT calculations of novel mixed ligand of copper complexes. *Appl. Organomet. Chem.* **39** (6), e70220. 10.1002/aoc.70220 (2025).

[63] Vilar, S., Cozza, G. & Moro, S. Medicinal chemistry and the molecular operating environment (MOE): application of QSAR and molecular Docking to drug discovery. *Curr. Top. Med. Chem.* **8** (18), 1555–1572. 10.2174/156802608786786624 (2008). 19075767 10.2174/156802608786786624

[64] Jejurikar, B. L. & Rohane, S. H. Drug designing in discovery studio. *(Book/Conference reference – no journal abbreviation available)* (2021).

[65] Elsayed, G. H. & Fahim, A. M. Studying the impact of Chitosan salicylaldehyde/Schiff base/CuFe2O4 in PC3 cells via theoretical studies and Inhibition of PI3K/AKT/mTOR signalling. *Sci. Rep.* **15** (1), 4129. 10.1038/s41598-025-01234 (2025). 39900661 10.1038/s41598-025-86096-7 PMC11790862

[66] Baildya, N. et al. Comparative study of the efficiency of silicon carbide, Boron nitride and carbon nanotube to deliver cancerous drug, azacitidine: A DFT study. *Comput. Biol. Med.* **154**, 106593. 10.1016/j.compbiomed.2023.106593 (2023). 36746115 10.1016/j.compbiomed.2023.106593

[67] Mandal, M. et al. Inhibitory efficacy of RNA virus drugs against SARS-CoV-2 proteins: an extensive study. *J. Mol. Struct.* **1234**, 130152. 10.1016/j.molstruc.2021.130152 (2021). 33678903 10.1016/j.molstruc.2021.130152 PMC7909904

[68] Fahim, A. M. & Shalaby, M. A. Synthesis, biological evaluation, molecular Docking and DFT calculations of novel benzenesulfonamide derivatives. *J. Mol. Struct.* **1176**, 408–421. 10.1016/j.molstruc.2018.08.072 (2019).

[69] Chattaraj, P. K., Cedillo, A. & Parr, R. G. Chemical softness in model electronic systems: dependence on temperature and chemical potential. *Chem. Phys.* **204** (2–3), 429–437. 10.1016/0301-0104(95)00388-8 (1996).

[70] Gordy, W. & Thomas, W. O. Electronegativities of the elements. *J. Chem. Phys.* **24** (2), 439–444. 10.1063/1.1742572 (1956).

[71] Hanna, A. & Tinkham, M. Variation of the coulomb staircase in a two-junction system by fractional electron charge. *Phys. Rev. B*. **44** (11), 5919–5922. 10.1103/PhysRevB.44.5919 (1991). 10.1103/physrevb.44.5919 9998444

[72] Parr, R. G. & Pearson, R. G. Absolute hardness: companion parameter to absolute electronegativity. *J. Am. Chem. Soc.* **105** (26), 7512–7516. 10.1021/ja00364a005 (1983).

[73] Domingo, L. R. et al. Quantitative characterization of the global electrophilicity power of common diene/dienophile pairs in Diels–Alder reactions. *Tetrahedron* **58** (22), 4417–4423. 10.1016/S0040-4020(02)00410-6 (2002).

[74] Vela, A. & Gazquez, J. L. A relationship between the static dipole polarizability, the global softness, and the Fukui function. *J. Am. Chem. Soc.* **112** (4), 1490–1492. 10.1021/ja00160a002 (1990).

[75] Ino, A. et al. Chemical potential shift in overdoped and underdoped La₂–xSrxCuO₄. *Phys. Rev. Lett.* **79** (11), 2101–2104. 10.1103/PhysRevLett.79.2101 (1997).

[76] Abdel-Maksoud, G., Fahim, A. M. & Sobh, R. A. Preliminary evaluation of green terpolymer of nano poly(methyl methacrylate/dimethylaminoethyl methacrylate/acrylamide) for the consolidation of bone artifacts. *J. Cult. Herit.* **73**, 139–149. 10.1016/j.culher.2025.01.002 (2025).

[77] Fahim, A. M. Exploring novel benzene sulfonamide derivatives: synthesis, ADME studies, anti-proliferative activity, Docking simulation, and theoretical investigations. *J. Indian Chem. Soc.* **101** (8), 101211. 10.1016/j.jics.2024.101211 (2024).

[78] Fahim, A. M., Magar, H. S. & Mahmoud, N. H. Synthesis, antimicrobial, antitumor activity, Docking simulation, theoretical studies, and electrochemical analysis of novel Cd(II), Co(II), Cu(II), and Fe(III) complexes containing barbituric moiety. *Appl. Organomet. Chem.* **37** (4), e7023. 10.1002/aoc.7023 (2023).

[79] Lu, T., Chen, F. & Multiwfn A multifunctional wavefunction analyzer. *J. Comput. Chem.* **33** (5), 580–592. 10.1002/jcc.22885 (2012). 22162017 10.1002/jcc.22885

[80] Tolan, H. E. M. et al. New mercaptopyrimidine derivatives synthesized with expected antimicrobial and antioxidant properties and theoretical study. *J. Mol. Struct.* **1324**, 140795. 10.1016/j.molstruc.2025.140795 (2025).

[81] Fahim, A. M., Dacrory, S. & Elsayed, G. H. Anti-proliferative activity, molecular genetics, Docking analysis, and computational calculations of uracil cellulosic aldehyde derivatives. *Sci. Rep.* **13** (1), 14563. 10.1038/s41598-023-41292 (2023). 37666882 10.1038/s41598-023-41528-0 PMC10477303

[82] Fahim, A. M. & Abas, K. M. Properties and computational insights of catalysts based on amide linked polymer for photo-Fenton remediation of Rhodamine B dye. *Sci. Rep.* **15** (1), 30566. 10.1038/s41598-025-30566 (2025). 40835870 10.1038/s41598-025-13192-z PMC12368231

